# Crucial Roles of Abscisic Acid Biogenesis in Virulence of Rice Blast Fungus *Magnaporthe oryzae*

**DOI:** 10.3389/fpls.2015.01082

**Published:** 2015-12-01

**Authors:** Carla A. Spence, Venkatachalam Lakshmanan, Nicole Donofrio, Harsh P. Bais

**Affiliations:** ^1^Department of Biological Sciences, University of DelawareNewark, DE, USA; ^2^Delaware Biotechnology InstituteNewark, DE, USA; ^3^Department of Plant and Soil Sciences, University of DelawareNewark, DE, USA

**Keywords:** *Magnaporthe oryzae*, rice blast, *Pseudomonas*, ABA, ISR

## Abstract

Rice suffers dramatic yield losses due to blast pathogen *Magnaporthe oryzae*. *Pseudomonas chlororaphis* EA105, a bacterium that was isolated from the rice rhizosphere, inhibits *M. oryzae*. It was shown previously that pre-treatment of rice with EA105 reduced the size of blast lesions through jasmonic acid (JA)- and ethylene (ETH)-mediated ISR. Abscisic acid (ABA) acts antagonistically toward salicylic acid (SA), JA, and ETH signaling, to impede plant defense responses. EA105 may be reducing the virulence of *M. oryzae* by preventing the pathogen from up-regulating the key ABA biosynthetic gene *NCED3* in rice roots, as well as a β-glucosidase likely involved in activating conjugated inactive forms of ABA. However, changes in total ABA concentrations were not apparent, provoking the question of whether ABA concentration is an indicator of ABA signaling and response. In the rice-*M. oryzae* interaction, ABA plays a dual role in disease severity by increasing plant susceptibility and accelerating pathogenesis in the fungus itself. ABA is biosynthesized by *M. oryzae*. Further, exogenous ABA increased spore germination and appressoria formation, distinct from other plant growth regulators. EA105, which inhibits appressoria formation, counteracted the virulence-promoting effects of ABA on *M. oryzae*. The role of endogenous fungal ABA in blast disease was confirmed through the inability of a knockout mutant impaired in ABA biosynthesis to form lesions on rice. Therefore, it appears that EA105 is invoking multiple strategies in its protection of rice from blast including direct mechanisms as well as those mediated through plant signaling. ABA is a molecule that is likely implicated in both tactics.

## Introduction

Rice (*Oryza sativa*) is a staple food crop world-wide, providing about one fifth of the calories consumed by humans. One of the largest problems impacting rice production is crop loss due to blast disease, caused by the hemibiotrophic fungal pathogen, *Magnaporthe oryzae*. Previously, we isolated and characterized a natural rice rhizospheric bacterium, EA105, that shows a strong direct antagonism toward *M. oryzae* vegetative growth and pathogenesis. When EA105 is root-inoculated on rice plants, it also triggers Induced Systemic Resistance (ISR) resulting in smaller blast lesions (Spence et al., 2014a), facilitated through jasmonic acid (JA) and ethylene (ETH) but not salicylic acid (SA) signaling (Spence et al., 2014b). Although SA, JA, and ETH are the three hormones that are most closely linked to plant defense, another important component is the stress responsive hormone, abscisic acid (ABA).

Abscisic acid is a small signaling molecule involved in multiple plant processes including seed dormancy, development, and response to abiotic and biotic stresses. Due to its involvement in numerous and overlapping processes, the activity of ABA is complex and tightly regulated at multiple steps. ABA is detected by a receptor complex, PYR/PYL/RCAR. The family of small soluble receptors termed PYR/PYL were identified using a synthetic ABA agonist, pyrabactin, and were named Pyrabactin Resistance 1 (PYR) and PYR1-Like (PYL). Within this family, some of the proteins were identified concurrently and also given the name Regulatory Components of ABA Receptors, or RCAR (Santiago et al., 2012). In the absence of ABA, PYR/PYL/RCAR receptors are found dimerized in the cytosol and nucleus. The binding of ABA results in dissociation of the dimers, and the monomer that is bound to ABA undergoes a conformational change forming a binding site for Phosphatase type 2 Cs (PP2Cs) (Santiago et al., 2012). The PP2Cs are inactivated when bound to the ABA-receptor complex. In the absence of ABA, PP2Cs inactivate SNF-1 Related Kinases (SnRK2s) which are positive regulators of ABA signaling (Szostkiewicz et al., 2010). When SnRK2s are no longer inhibited by PP2Cs, they enter the nucleus and phosphorylate targets such as *SLAC1, KAT1, AtRbohF*, thus activating, transcription factors which positively influence expression of stress/ABA-responsive genes (Kulik et al., 2011). These genes contain sequences within their promoters called ABA Responsive Elements (ABREs) that are recognized by transcription factors, referred to more specifically as ABRE-binding proteins (AREBs) or ABRE binding factors (ABFs) (Fujita et al., 2013).

The amount of ABA in a particular location within a plant tissue depends on biosynthesis, catabolism, transport, and compartmentalization (Ye et al., 2012). In plants, ABA is synthesized through the cleavage of carotenoids in a multi-step pathway (Schwartz et al., 1997, 2003). The key regulatory enzyme in ABA biosynthesis is coded for by Nine-*cis*-epoxycarotenoid dioxygenase 3 (*NCED3*) (Liotenberg et al., 1999; Qin and Zeevaart, 1999). The first committed step in ABA catabolism is hydroxylation of the methyl group at the eighth carbon position. ABA contains three methyl groups, that can be hydroxylated, with C8 hydroxylation being most closely linked to catabolism. Hydroxylation does not inactivate ABA, but it can flag ABA for conversion into phaseic acid (PA) and subsequently dihydrophaseic acid (Ye et al., 2012). In rice, there are three enzymes with differing expression patterns that hydroxylate ABA, *OsABA8ox1*, *2*, and *3* (Ye et al., 2012) and are homologs of *Arabidopsis thaliana CYP707A* (Nambara and Marion-Poll, 2005). *OsABA8ox1* is primarily responsible for ABA catabolism following drought stress, and is negatively regulated by ethylene (Saika et al., 2007). Additionally, ABA can be reversibly inactivated through conjugation, primarily to glucosyl esters forming ABA-GE that can be stored in vacuoles or apoplasts. The addition of glucosyl esters to ABA is catalyzed by an ABA glucosyl transferase (Xu et al., 2002) and the conjugate can be subsequently removed through hydrolysis by a β-glucosidase such as *AtBG1* (Lee et al., 2006). Glucosyl transferases and β-glucosidases involved in the inactivation and activation of ABA have not previously been characterized in rice.

While ABA is a crucial molecule for regulating plant growth, development, and stress response, some phytopathogens have evolved mechanisms to stimulate overproduction of ABA in plants, resulting in the suppression of Systemic Acquired Resistance (SAR), while also causing a reduction in growth, transpiration, and photosynthesis (Loake and Grant, 2007). SAR is typically mediated through SA signaling, and ABA acts antagonistically to SA signaling, blocking the SAR response (Jiang et al., 2010; Xu et al., 2013; Meguro and Sato, 2014). It has been shown multiple times that ABA suppresses not only SA-mediated defense signaling but also JA and ETH-mediated defense signaling (Anderson et al., 2004; Adie et al., 2007; Ton et al., 2009; Atkinson and Urwin, 2012; Nahar et al., 2012). Elevated ABA levels in rice plants are associated with increased disease severity of rice blast caused by *M. oryzae* (Koga et al., 2004; Jiang et al., 2010; Yazawa et al., 2012) as well as bacterial blight caused by *Xanthomonas oryzae* (Xu et al., 2013). The reverse highlighted the same point; knocking down ABA levels reduces susceptibility to blast by impairing the ability of *M. oryzae* to penetrate host cells, ultimately resulting in reduced disease symptoms (Yazawa et al., 2012).

In addition to increasing plant susceptibility to disease, we hypothesize that high levels of ABA in fungi may also directly promote virulence in *M. oryzae*. ABA biosynthesis and signaling is likely to be an ancient process found in early unicellular eukaryotes which has followed divergent evolution (Hauser et al., 2011). Several phytopathogens retain the ability to synthesize ABA, though the role of fungal-derived ABA is still unclear and there is evidence that most fungal-produced ABA is secreted (Hartung, 2010). The fungal ABA biosynthesis pathway is distinct from that of plants. The ABA biosynthesis gene cluster in fungi was first identified in *Botrytis cinerea* (Siewers et al., 2006) and has been named the “direct” or “mevalonate” pathway in contrast to plants that cannot use mevalonic acid as a precursor to ABA biosynthesis (Hirai et al., 2000). ABA perception and signaling mechanisms have also diverged, with differences arising even between monocots and dicots (Hauser et al., 2011). ABA production has been documented in *M. oryzae* races 007.0 and 102.0 (Jiang et al., 2010) though it has not previously been shown in the sequenced reference strain, 70-15. We have examined the role of ABA in a three-way communication between rice, the fungal pathogen *M. oryzae*, and a natural rice beneficial bacterial isolate with the goal of further elucidating the mechanisms by which this bacterium can directly antagonize *M. oryzae* and trigger ISR in rice to protect against blast.

## Materials and Methods

### Fungal and Bacterial Strains and Growth Conditions

Wild type *M. oryzae* 70-15, the sequenced reference strain, was used throughout the experiments. For vegetative growth, the fungi were placed on complete medium (CM) containing sucrose (10 g/L), casamino acids (6 g/L), yeast extract (6 g/L), and 1 mL of *Aspergillus nidulans* trace elements (Per 100 mL: 0.22 g MnSO_4_⋅H_2_0, 0.05 g KI, 0.02 g ZnSO_4_⋅7H_2_0, 0.01 g H_3_BO_4_, 0.1 mL concentrated H_2_SO_4_, 0.008 g NiCl_2_⋅6H_2_0, 0.007 g CoCl_2_⋅6H_2_0). Oatmeal agar consisting of ground oats (50 g/L) and agar (15 g/L) were used for sporulation. Plates were kept at 25°C with constant fluorescent light. Bacterial strains EA105, EA106, and EA201 were isolated from rhizospheric soil surrounding the roots of rice cultivar M-104 grown in the field by Dr. Venkatesan Sundaresan’s lab from the University of California (Davis). The bacteria were cultured in liquid or solid Luria Bertani (LB) medium at 28–30°C.

### Plant Materials and Growth Conditions

*Oryza sativa* cultivar M-104 was donated by Dr. Venkatesan Sundaresan from the University of California (Davis). Hyper-susceptible genotype Seraceltik was used for *M. oryzae* infections. Rice seeds were dehusked and sterilized using ethanol and bleach. Ten-day-old seedlings were transferred to sterile clear plastic boxes containing 50 mL of Hoagland’s medium, pH 5.7 and placed on a shaker at 80 rpm. Treatments were done at 14 days.

### Rice Treatments

Exponential phase bacterial cultures were washed in water and re-suspended to approximately 1 × 10^9^ cells per mL. In each clear box containing rice seedlings, 50 μL of bacteria was added to the media, for a final concentration of 1 × 10^6^ cells per mL. For 70-15 treatment, spores were grown on oatmeal, then scraped into sterile water and filtered through a sterile miracloth (EMD Millipore). Rice leaves were dipped in a spore suspension containing 0.2% gelatin and 1 × 10^5^ spores per mL for 5 min. In the tri-trophic interaction, spores were added 24 h after bacterial treatment. For growth hormone treatments, each was added into the liquid media at the following concentrations: ABA (100 μM), IAA (20 μM), IBA (1 μM), Kinetin (100 μM), GA (50 μM). Kinetin stock was prepared in 1N NaOH, while ABA, IAA, IBA, and GA stocks were prepared in methanol. All were filter sterilized. Controls were treated with an equal amount (1 μl per mL) of methanol. Each treatment was done in biological triplicate, and each biological replicate contained five plants.

### *In Planta* Infection Assays

Rice plants of cultivars M-104, Nipponbare, and Seraceltik were grown in soil for 3 weeks. To check the effect of bacterial priming, overnight cultures of bacteria were washed in water and re-suspended to 0.5 OD. For each plant, 2 mL of bacteria were dispensed onto the soil surface at the base of the plant. At 24 h post bacterial treatment, the second youngest leaf was cut and affixed to a large 15 cm diameter petri dish, on top of moistened paper towels and treated with 70-15 spores. Spores were grown on oatmeal agar for 10 days, and were subsequently scraped into sterile water with 0.2% gelatin. Spore concentration was adjusted to 10^5^ spores/mL. On each leaf, a total of 4–30 μL droplets of spores were placed along the length. Plates were incubated in the dark for 24 h at 25°C, after which time the spore droplets were wicked away. Plates were then kept in cycles of 16 h light/8 h darkness for 5 days at 25°C. On the fifth day, the length and width of lesions were measured. A minimum of eight leaves with four droplets per leaf were included per replicate. Three biological replicates were completed.

### Spore Germination and Appressoria Formation Assays

Plastic coverslips were sterilized with ethanol and UV, and used as a hydrophobic surface to encourage spore germination and appressoria formation. *M. oryzae* spores were grown on oatmeal agar for 10 days prior to being scraped into water and filtered through a miracloth. Each coverslip was inoculated with a 50 μL drop containing a final concentration of 10^5^ spores/mL, and/or 10^5^ bacterial cells/mL (also in water). The coverslips were placed in petri dishes with wet filter disks in the center to promote humidity. Plates were sealed and placed in the dark. Germination percentages were calculated after 2 h incubation, and appressoria formation was determined after 6 h. Coverslips were imaged using a Zeiss Axioscope2 light microscope. Three images were taken per coverslip, and five coverslips were used per treatment. Three biological replicates were examined.

### ABA Quantification

Plants and spores were treated as described above. Roots, shoots, and fungal tissue were ground in liquid nitrogen. For plant samples and spore samples, approximately 100 mg of tissue was used. For fungal mycelial samples, approximately 250 mg of tissue was used. ABA was extracted from each sample in 1 mL of 90% methanol containing 10 mg of butylated hydroxytoluene and 20 mL of glacial acetic acid per liter. The extraction was done at 4°C for 24 h. The samples were then used with the Phytodetek Abscisic Acid ELISA-based kit, per the directions. Plant samples were run at the following dilutions (in TBS buffer): 1:10, 1:20, 1:40, 1:80, and 1:160. Fungal samples were run at the following dilutions: 1:10, 1:100, 1:200, and 1:400. ABA content was calculated per gram of tissue. Each biological replicate consisted of five plants or five fungal plates, and the experiment was done in biological triplicate.

### Gene Expression

Tissue was ground in liquid nitrogen and RNA was extracted using the EZ-10 Total RNA Mini-prep kit (BioBasic). Samples were treated with DNaseI (Thermo Scientific) and cDNA was synthesized from 500 ng of RNA using the High Capacity cDNA Reverse Transcription kit (Applied Biosystems). Standard Taq polymerase (New England Biolabs) was used for PCR, and products were run on a 1.4% agarose gel. Gene-specific primers are listed in Supplementary Table S2. Band intensities were quantified using ImageJ, and normalized to ubiquitin expression. Each biological replicate was pooled from 5 plants, and each treatment was done in biological triplicate.

### Constructing Mutants in *M. oryzae* 70-15

Adaptamer-mediated PCR (Reid et al., 2002), a method based on homologous recombination, was used to create knock out mutants in strain 70-15. Briefly, genes of interest were replaced with a hygromycin resistance cassette. For each gene to be knocked out, a 1.2 kb segment upstream of the 5′ UTR and another 1.2 kb segment downstream of the 3′ UTR was amplified. Adaptors were added to the 3′ end of the first segment and the 5′ end of the second segment. The hygromycin resistance cassette was amplified from plasmid pCB1003 using primers that had adaptors complementary to those used in amplifying the upstream and downstream segments. All three segments were combined in a PCR reaction to make the full-length constructs, approximately 3.3 kb. Creation of protoplasts and transformations were conducted following traditional methods (Sweigard et al., 1992). Gene disruption mutants were identified by PCR screening using primers outside the flanking regions and gene specific primers and further confirmed by Southern blot analysis. Primers are listed in Supplementary Table S3.

### Southern Hybridization

Total DNA of *M. oryzae* 70-15 and mutants was isolated using Qiagen DNeasy plant mini kit (Qiagen, Inc.,) and digested with restriction enzymes, electrophoresed on 1.0% agarose gels in 1 × TAE buffer, and transferred onto Whatman Nytran SPC nylon membrane (Whatman, Inc.,) overnight by capillary action. DIG labeled DNA probes of HPH were generated using a DIG Probe Synthesis Kit (Roche Diagnostics Corporation, Indianapolis, IN, USA), hybridized to blots overnight at 48°C, washed under high stringency conditions and exposed to Kodak Biomax light film. Procedures recommended by the manufacturer of the kits were used. Although we are aware of its utility, a complemented line for the ABA4 mutant was not created in this study due to time and resource limitations. However, our experiments with exogenously applied ABA provide strong proof that the phenotypes observed are related to the deletion of the ABA4 gene.

### Sporulation Assay

A 5 mm plug of spores was plated in the center of an oatmeal agar plate. Plates were kept under fluorescent light with a cycle of 16 h light/ 8 h dark at room temperature. After 7 days, the surface of the plate was scraped to collect the spores, which were put into 1 mL of water. The spores were then counted using a haemocytometer. For each strain, there were three biological replicates, done in technical quadruplicate.

### Statistical Analysis

Statistical analyses of the results were performed using the statistical software JMP 10. To compare across treatments, the Tukey’s HSD test was used and results were considered to be statistically different when *p* < 0.05.

## Results

### EA105 May Prevent 70-15 Spores from Increasing ABA Biosynthesis and Signaling in Rice

**FIGURE 1:**
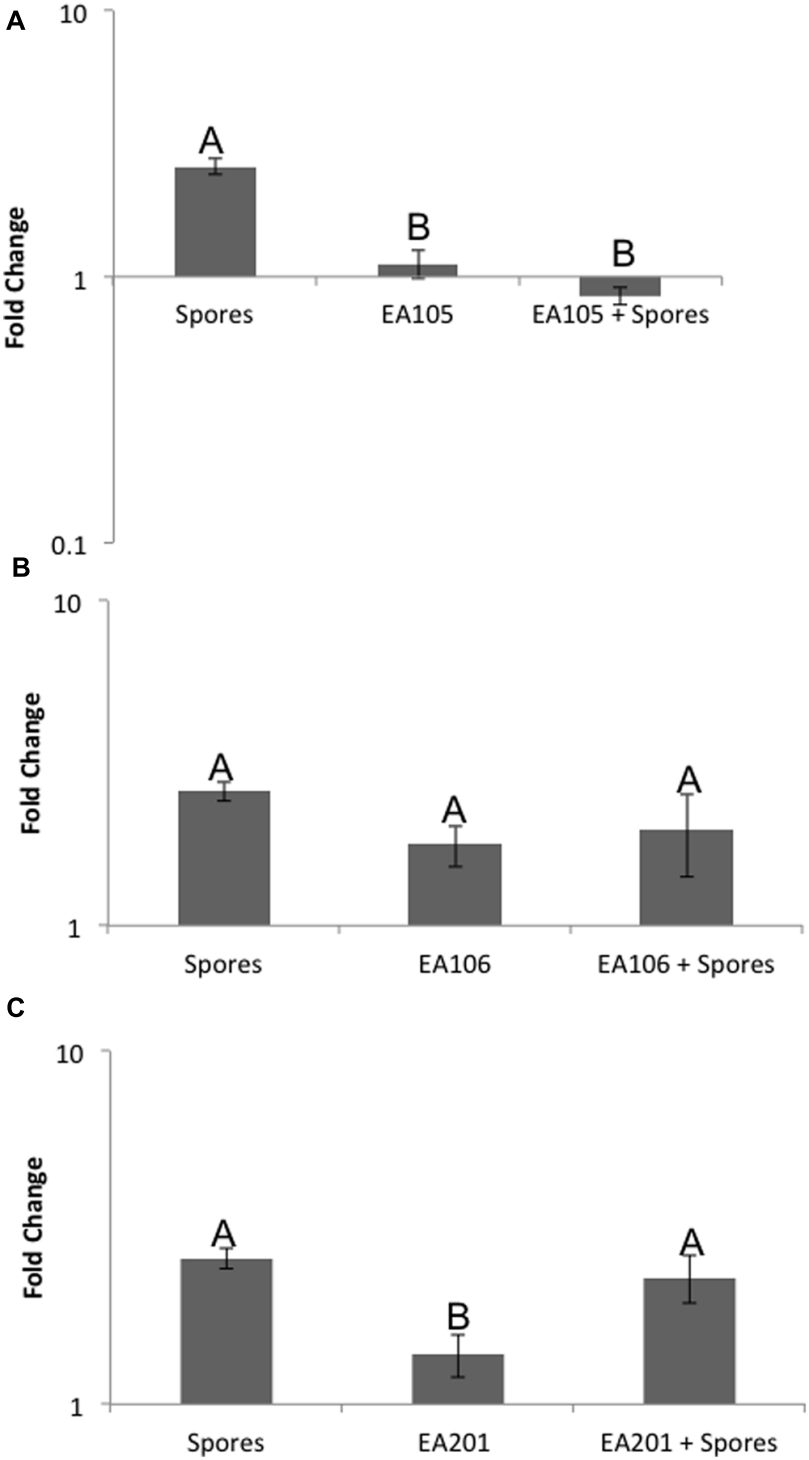
**Expression of ABA biosynthesis gene, *NCED3*.** Roots were inoculated with either **(A)** EA105, **(B)** EA106, or **(C)** EA201, 24 h prior to being exposed to spores. Error bars indicate standard error based on three biological replicates, each including five plants. Different letters represent a statistically significant difference based on the Tukey-Kramer test, *p* < 0.05.

**FIGURE 2:**
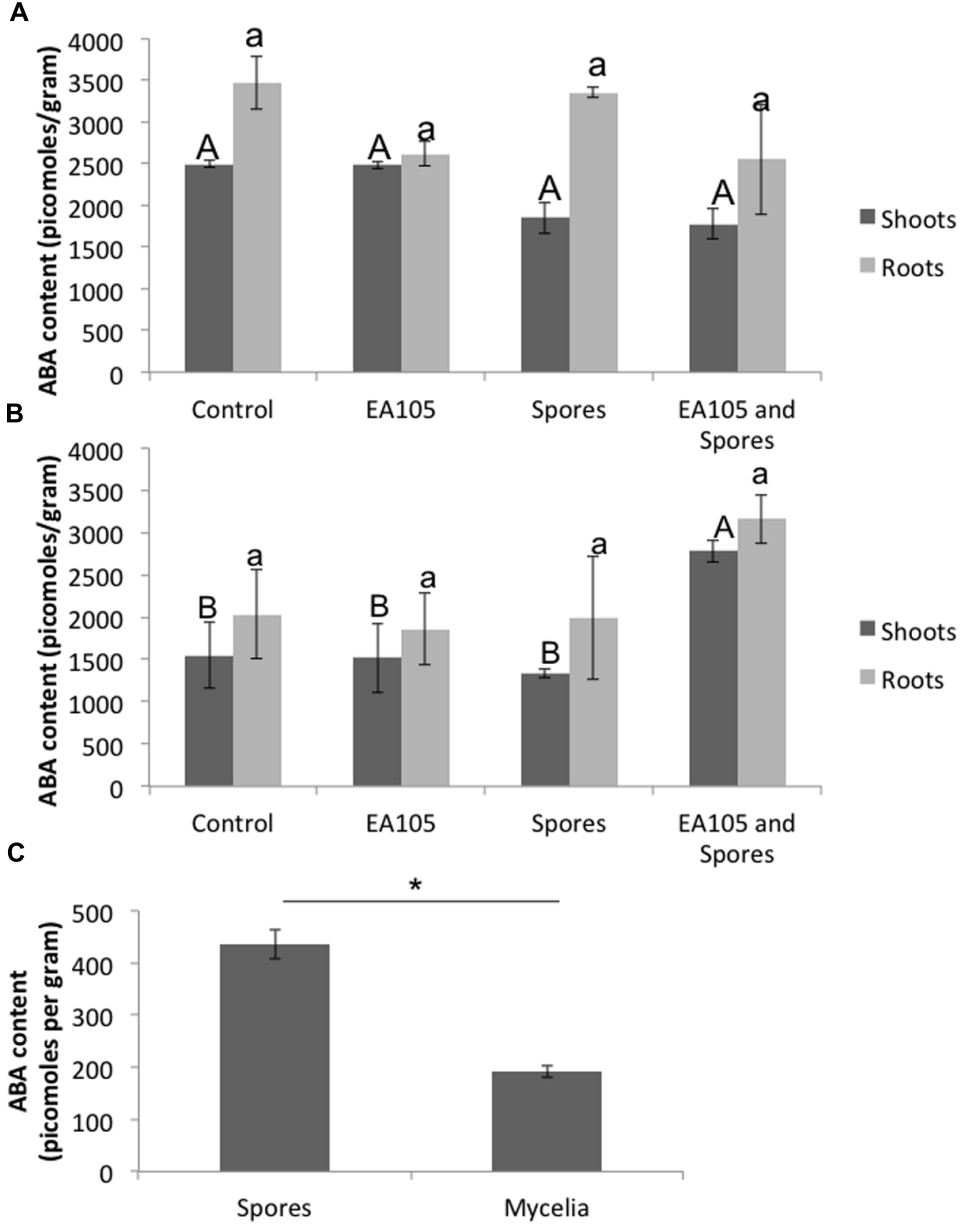
**Abscisic acid (ABA) concentrations in rice roots and shoots, as well as 70-15 spores and mycelia, using an ELISA-based kit.** Root and shoot tissue was collected separately at **(A)** 24 h post 70-15 treatment and **(B)** 48 h post 70-15 treatment for ABA extraction. **(C)** ABA content was quantified in untreated 70-15 spores and mycelia. Error bars indicate standard error based on three biological replicates, each pooled from 5 plants, or 3 plates of fungi. Each replicate was quantified in quadruplicate. Different letters represent a statistically significant difference based on the Tukey-Kramer test when *p* < 0.05. For spores and mycelia, mean ABA concentration is statistically significant, *p* = 0.0036, based on a *t*-test.

Previously, we showed that when *Pseudomonas chlororaphis* EA105 (hereafter EA105) was inoculated onto uninfected rice plants, there was approximately a 10-fold increase in the ETH responsive genes *EIL1* and *ERF1* as well an approximately threefold increase in the JA responsive genes *JAR1* and *WRKY30* at 24 h post treatment (Spence et al., 2014b). However, the SA responsive genes *PR1* and *WRKY77* were minimally affected (Spence et al., 2014b). From that data, it was concluded that isolate EA105 induces systemic resistance in rice against blast in an ETH and JA dependent manner (Spence et al., 2014b). Since elevated ABA levels are associated with increased susceptibility, we examined the expression of *NCED3*, the rate-limiting enzyme involved in ABA biosynthesis, in roots where *NCED3* is most active. 70-15 spores up-regulated *NCED3* while EA105 did not affect its expression. Interestingly, 70-15 spores were unable to induce *NCED3* expression in plants that were pre-treated with EA105 (**Figure 1A**). For comparison, two other isolates recovered from the same soil sample as EA105 were also tested (Spence et al., 2014a). Isolate EA201 inhibits fungal diameter *in vitro* but root treatment does not reduce lesions. Contrastingly, EA106 has no direct antifungal capabilities, but does induce resistance when treated at the root surface. EA105 is distinct from both, because it can directly inhibit fungi and also induce resistance against *M. oryzae* (Spence et al., 2014a). Pretreatment with rice isolates EA106 and EA201 did not prevent 70-15 from up-regulating *NCED3* (**Figures 1B,C**). To see if *NCED3* up-regulation coincided with higher ABA levels, total ABA concentrations in roots and shoots treated with bacteria, fungus, or both were examined. However, there were no significant differences in the ABA content (**Figure 2A**). In all treatments, there were approximately 2000–2500 picomoles of ABA per gram of plant tissue. ABA content was checked at the same time-point used for expression analysis as well as 24 h later, and still no differences were apparent (**Figure 2B**). At the second time-point, there was actually a slight increase in ABA levels in plants that were treated with both EA105 and spores (**Figure 2B**). ABA concentrations were also determined in 70-15 spores and mycelia. We found that ABA is produced by 70-15, with mycelia producing around 200 picomoles per gram and spores producing more than 400 picomoles per gram (**Figure 2C**).

Since the ABA concentrations did not match what was seen with *NCED3* expression patterns, it was necessary to examine other steps at which ABA signaling is regulated. We designed primers for multiple genes related to ABA including those involved in catabolism, perception, signaling, and response, to gain a better understanding of how EA105 and 70-15 spores are affecting ABA signals in rice. The expression of *OsABA8ox1*, involved in ABA catabolism in rice, was largely unaffected by any of the treatments; however, there was slightly less of an induction of *OsABA8ox1* in plants treated with spores or exogenous ABA (Supplementary Figures S1 and S2). Similarly, there were no significant expression changes in ABA receptor *RCAR5* (Supplementary Figures S1 and S2).

Abscisic acid activity can also be modulated through inactivation and activation by adding or removing, respectively, glucosyl esters. In rice, the genes involved in this process had not previously been characterized. A BLAST search of the protein sequence for *AtBG1*, a gene implicated in the activation of ABA in *A. thaliana*, revealed a putative rice β-glucosidase. This gene was expressed more strongly in shoots than roots, and its expression in shoots was induced by ABA (Supplementary Figure S2). When plants were treated with EA105 and ABA together, there was no longer up-regulation of this gene. Although the expression of this gene was weaker overall in roots, there was nearly 24-fold up-regulation in plants infected with 70-15 spores (Supplementary Figure S3). Similar to what was seen with *NCED3*, the presence of EA105 prevents spores from up-regulating this ABA-activating gene in roots. In *A. thaliana*, the inactivation of ABA is typically catalyzed by *UGT71B6*, a UDP glucosyl transferase. Using a protein BLAST of this gene, a putative rice glucosyl transferase was found. Expression of this gene in roots was very low compared to shoots. Interestingly, shoot expression patterns mimicked those of the β-glucosidase, with ABA inducing this gene more than 10-fold while EA105 + ABA eliminated the induction of this gene (Supplementary Figure S2).

Moving further through the ABA signaling pathway, the expression of Mitogen-activated protein (MAP)-kinase *OsMPK1* was also examined, but showed minimal expression changes (Supplementary Figure S1). When ABA signaling is able to proceed, it culminates in the expression of genes containing ABREs in their promoter regions. One such gene of many is *Rab25*, although its expression was not affected by the treatments (Supplementary Figure S2).

### Exogenous ABA Promotes Virulence in *M. oryzae*

**FIGURE 3:**
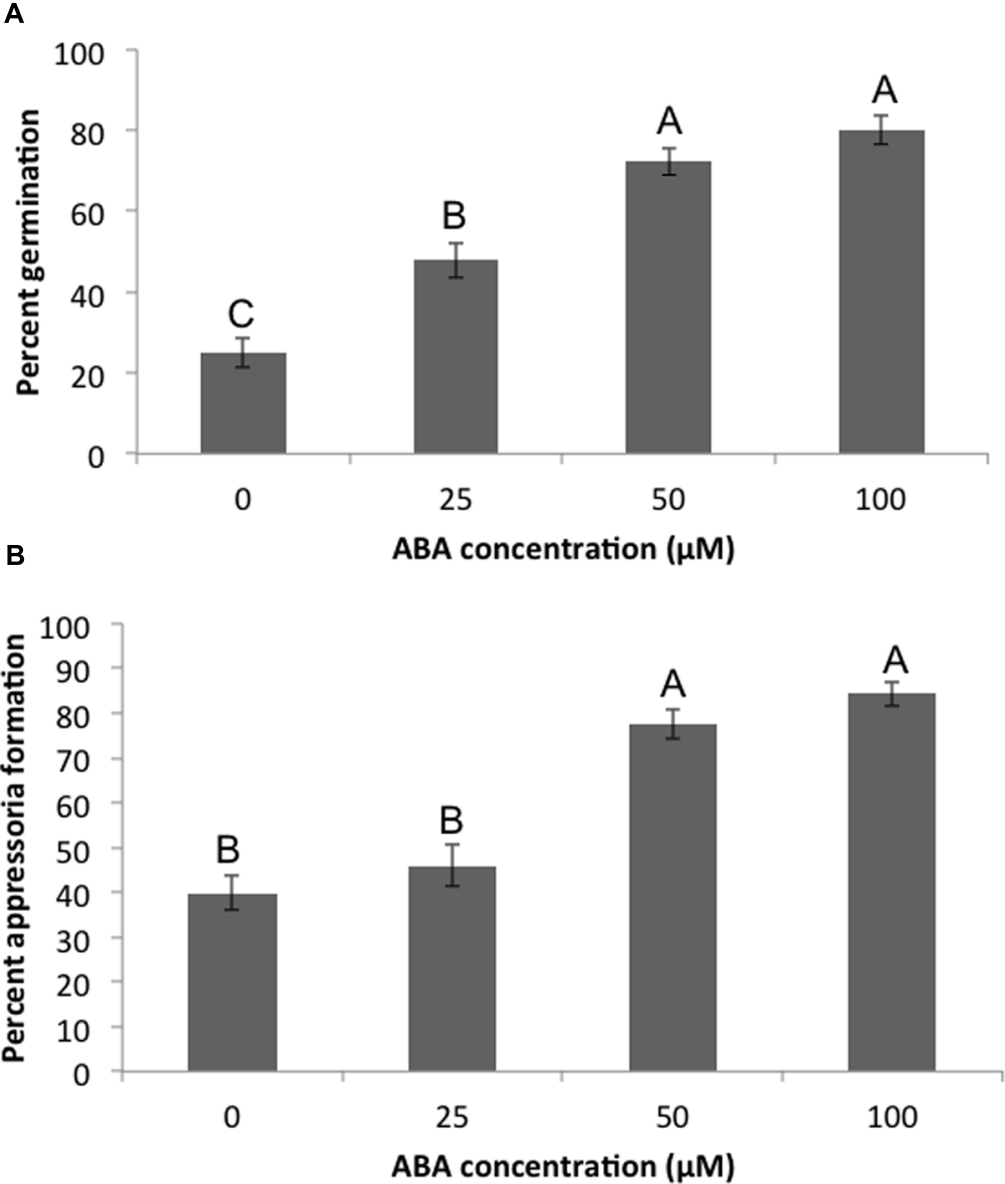
***Magnaporthe oryzae* 70-15 spores treated with different concentrations of ABA.** Images were taken at 2 and 6 h post-treatment and the percentage of spores **(A)** germinating or **(B)** forming appressoria were quantified. This experiment was repeated three times with five coverslips per treatment and three images per coverslip. Different letters represent statistical significance based on the Tukey-Kramer test (*p* < 0.05).

**FIGURE 4:**
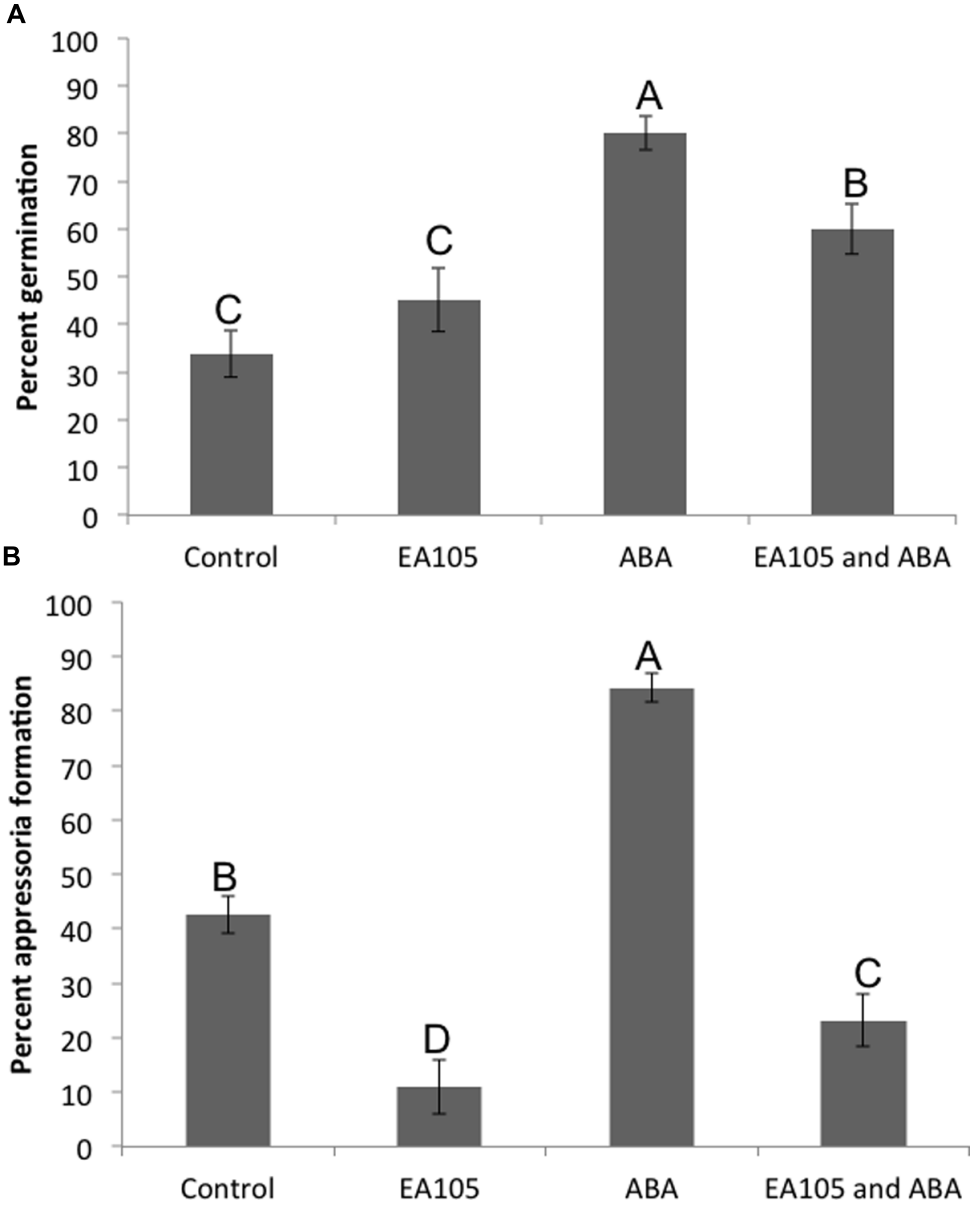
***Magnaporthe oryzae* 70-15 spores treated with EA105 and/or exogenous ABA. (A)** Percent germination was quantified at 2 h post treatment and **(B)** percent appressoria formation was quantified at 6 h post treatment. The experiment was repeated three times with five coverslips per treatment and three images per coverslip. Different letters represent statistical significance based on the Tukey-Kramer test (*p* < 0.05).

Pathogenesis of *M. oryzae* begins with spore germination. The crucial step in virulence is the formation of the appressorium, a specialized infection structure that accumulates high turgor pressure and forms a penetration peg that can enter the rice cuticle. To test whether ABA could enhance pathogenicity in *M. oryzae*, 70-15 spores were treated with ABA ranging from 10 to 100 μM. By 3 h almost all spores germinated in the untreated controls, and by 24 h most formed appressoria. To see if ABA was accelerating these processes, germination was examined at 2 h and the initiation of appressoria formation was examined at 6 h. Spores that had been exposed to 50 or 100 μM ABA had a higher percent germination than those that were not exposed to ABA at 2 h post treatment (**Figure 3**). Also, the percent of germinated spores that were forming appressoria at 6 h post treatment was higher in the presence of 50 or 100 μM ABA (**Figure 3**). In addition to ABA, other plant growth regulators were tested including gibberelic acid (GA), the natural auxin indole-3-acetic acid (IAA), a synthetic auxin indole-3-butryic acid (IBA), and the cytokinin, kinetin. Aside from ABA, only GA was able to increase percent germination at 2 h, but did not increase appressoria formation at 6 h (Supplementary Figure S4). Kinetin had no effect on germination, but increased appressoria formation at 6 h. The natural and synthetic auxins had no effect on *M. oryzae* 70-15 spore germination or appressoria formation (Supplementary Figure S4). The germination and appressoria formation was also quantified in spores treated with EA105 and ABA together. Previously, we have shown that EA105 had a minimal effect on spore germination at 3 h, but almost completely abolished appressoria formation in *M. oryzae* at 24 h (Spence et al., 2014a). At the earlier time points, EA105 behaves similarly. The percent germination is not statistically different from the control, while appressoria formation is greatly reduced (**Figure 4**). When EA105 and ABA are co-treated on spores, the percent germination is about half way between what was seen with ABA or EA105 treatment alone. The percent of spores that formed appressoria decreased from about 84% with ABA treatment alone to about 23% when treated with both EA105 and ABA together (**Figure 4**).

### Impairing ABA Biosynthesis in *M. oryzae* Dramatically Reduces Virulence

Although the genes responsible for ABA biosynthesis in *M. oryzae* have not been identified or characterized, we found hypothetical genes in 70-15 that were orthologous to those responsible for ABA biosynthesis in the fungal pathogen *Botrytis cinerea*. In *B. cinerea*, there are four biosynthesis genes (Siewers et al., 2006). Orthologs were found for all the genes except ABA3 (Supplementary Table S1). Based on the annotated *M. oryzae* 70-15 genome, we also found an ABA G-protein coupled receptor (GPCR) gene. All four genes are located on chromosome 3 in 70-15 (Supplementary Table S1; Supplementary Figure S5). To determine if these genes were functional, their expression was examined in 70-15 spores and mycelia. For all the genes, expression in spores was higher than in mycelia and ABA2 did not appear to be expressed at all in mycelia (Supplementary Figure S6). Expression was also examined in spores and mycelia that were treated with EA105, but only very subtle down-regulation of the genes occurred (Supplementary Figure S6). There were no significant changes in ABA content with EA105 treatment, though there was a slight but non-significant decrease in ABA content of mycelia treated with EA105 compared to untreated mycelia (Supplementary Figure S6).

**FIGURE 5:**
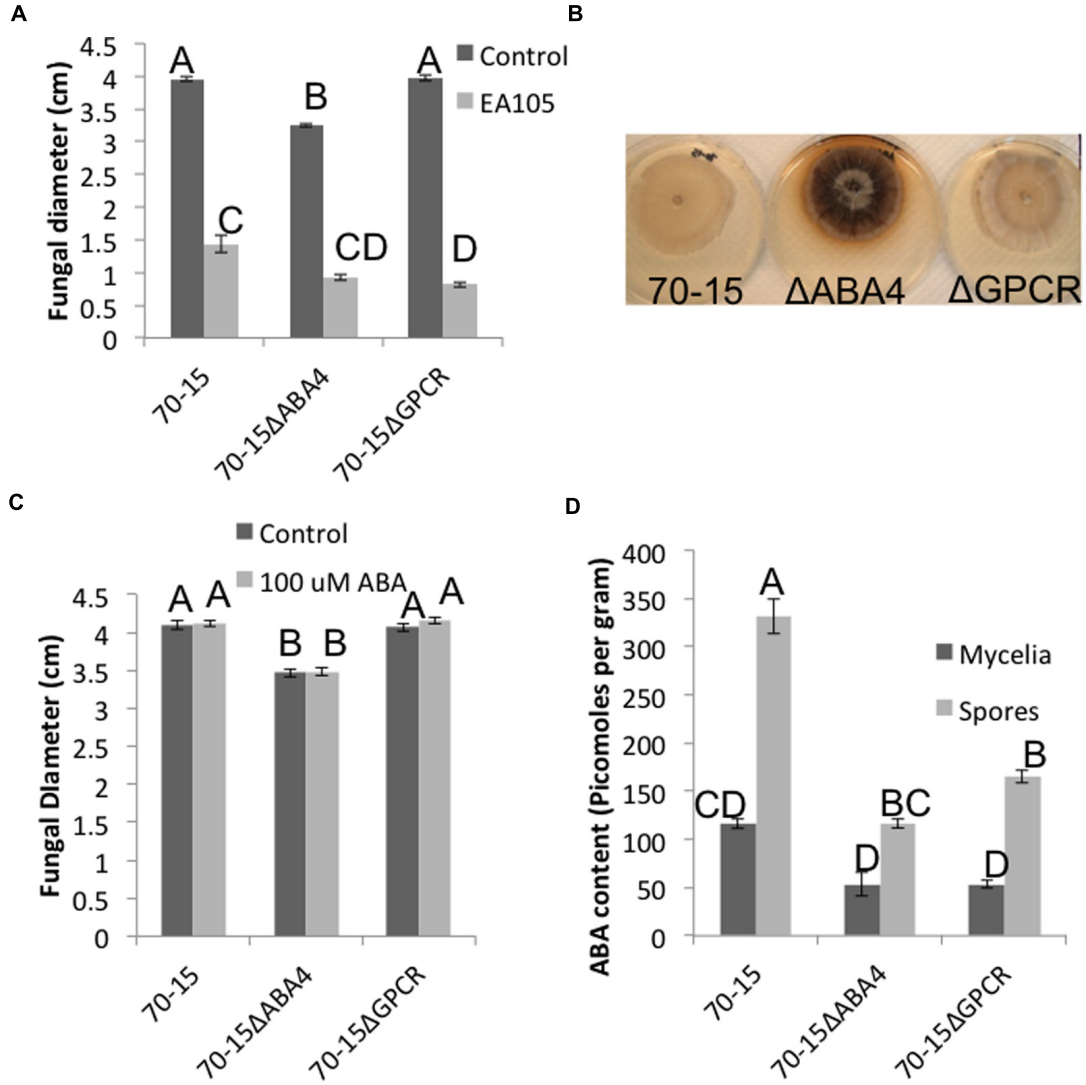
**Vegetative growth characteristics of ABA4 and ABA GPCR knockout mutants. (A)** Fungal diameter of 70-15, 70-15ΔABA4, and 70-15ΔGPCR at 7 days after culturing, the in presence and absence of EA105. **(B)** At 14 days post culturing, 70-15ΔABA4 looks strikingly different than its parental strain due to pigmentation. **(C)** Fungi were grown on CM amended with 100 μM ABA and fungal diameters were measured after 7 days. **(D)** ABA content was quantified in spores and mycelia of 70-15 and the two mutants. Different letters represent statistical significance based on the Tukey-Kramer test (*p* < 0.05).

**FIGURE 6:**
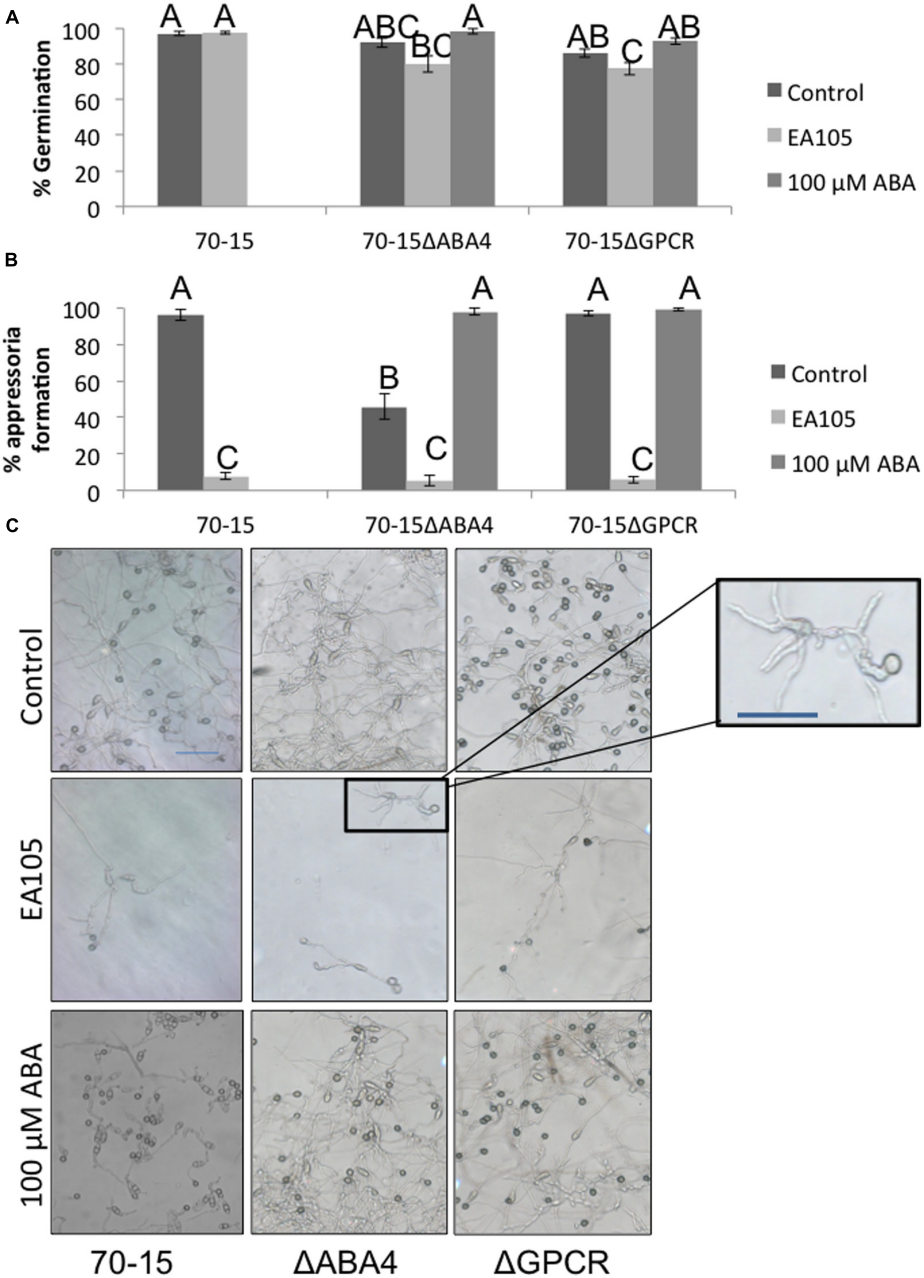
**Spore germination and appressoria formation in 70-15 and ABA mutants. (A)** Spore germination was measured 3 h after resuspension and incubation on a hydrophobic plastic coverslip. **(B)** Appressoria formation was measured at 24 h. **(C)** Light microscope images were taken in areas which were dense with spores (top row) as well as in sparse areas where individual spores could be discerned. An enlarged imaged of the ABA4 mutant is displayed to show the hyperbranching phenotype. Scale bars are 50 μM. Supplementation of ABA (100 μM) reverts the phenotype in ABA4 mutant. Different letters represent statistical significance based on the Tukey-Kramer test (*p* < 0.05).

An attempt was made to knock out ABA1, 2, and 4 in 70-15, however, potential ABA1 and ABA2 mutants were extremely slow growing and small, and we were unable to confirm that these were mutants. ABA4 was successfully knocked out, as well as the ABA GPCR (Supplementary Figure S5). The GPCR mutant largely resembled the parental 70-15, but the ABA4 mutant had distinct phenotypic differences in multiple stages of its life cycle. Vegetatively, the 70-15ΔABA4 mutant grew slower than 70-15 and never reached the same size (**Figure 5A**). Additionally, the mycelia became darkly pigmented beginning around 7 days after re-culturing and striking difference were apparent by 14 days (**Figure 5B**). Sporulation was also impaired in 70-15ΔABA4, while 70-15ΔGPCR produced more conidia than 70-15 (Supplementary Figure S7). The appearance of the 70-15ΔABA4 spores was also different than that of the wild-type, with unusual white patches visible on the spore plates (Supplementary Figure S7). Additionally, a very dark pigment was secreted into the media, and was left behind after spores were removed from the plates. There were not any significant differences in germination, although germination of both mutants was inhibited by EA105 treatment, which was not the case for 70-15 (**Figure 6A**). The 70-15ΔABA4 mutant was severely impaired in appressoria formation as compared to the other two strains (**Figure 6B**) and there were distinct phenotypic differences that could be seen microscopically. The ABA4 mutants showed hyper-branching of the germ tubes, as well as unusual bulges along the hyphae with less melainized appressoria (**Figure 6C**).

Abscisic acid was quantified in the mycelia and spores of both mutants and compared with parental 70-15. In mycelia, both mutants produced roughly half the ABA of 70-15. The total amount of ABA produced by spores was higher than that in mycelia. The ABA4 mutant spores produced slightly less than half the amount of ABA produced by wild type spores. Similarly, the GPCR mutant spores also produced approximately half the amount of ABA that was produced by wild-type (**Figure 5D**).

Interestingly, adding exogenous ABA to the media did not complement 70-15ΔABA4’s vegetative growth defect, nor did it effect growth of 70-15 or 70-15ΔGPCR (**Figure 5C**). However, adding exogenous ABA to spores enabled the ABA4 mutant to form as many appressoria as the parental strain (**Figure 6B**) and partially complemented the aberrant phenotype of hyperbranching and bulging.

**FIGURE 7:**
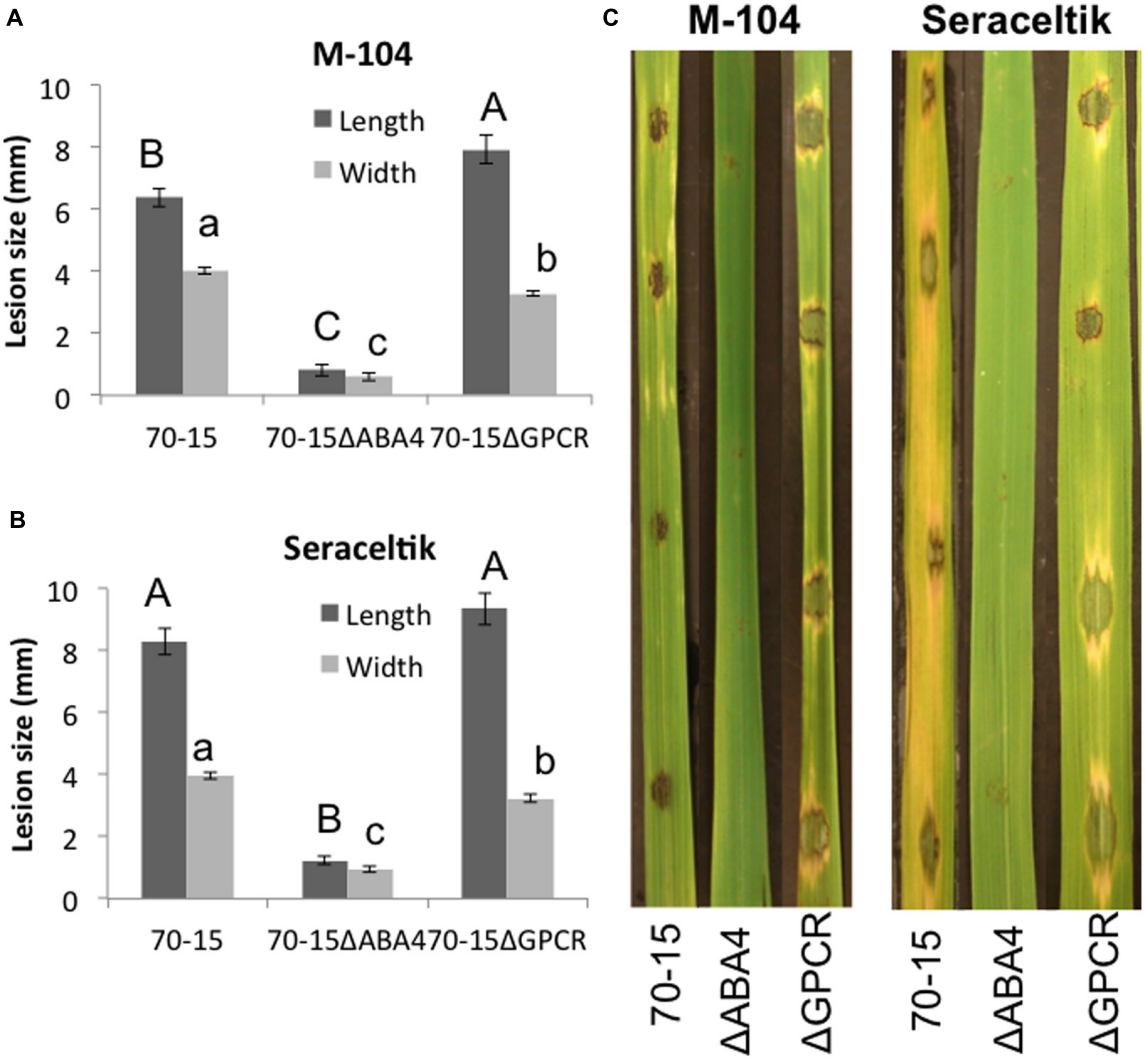
**Ability of ABA mutants to infect rice.** M-104 and Seraceltik rice plants were infected with 70-15, 70-15ΔABA4, or 70-15ΔGPCR and lesion length and width were measured. Photographs were taken 5 days after infection. Different letters represent statistical significance based on the Tukey-Kramer test (*p* < 0.05).

The mutants were also tested for their ability to form lesions on rice leaves. Strikingly, the 70-15ΔABA4 mutant was unable to properly infect. In many cases, the mutant did not have any effect on the leaf, or left a tiny black speck (**Figure 7**). The small spots that formed were much smaller than the lesions caused by 70-15 or 70-15ΔGPCR (**Figure 7**). When the experiment was extended for several more days, all of the leaves began drying and curling, and the lesions from 70-15 and 70-15ΔGPCR kept spreading, but the 70-15ΔABA4 spots did not grow. The mutants were also tested on plants that had been root-primed with EA105 for 24 h. EA105 retained the ability to reduce lesion size caused by 70-15 and 70-15ΔGPCR, but since 70-15ΔABA4 did not form lesions, there was no effect from EA105 priming. The same trend was apparent in rice cultivars M-104 and Seraceltik (Supplementary Figures S7–S9).

## Discussion

With the global population expected to exceed 9 billion by 2050, developing an effective and sustainable method to reduce crop loss from rice blast could have a significant impact on food security. We have identified natural rice rhizospheric isolate EA105, which has the ability to reduce the symptoms of blast in rice. However, another important aspect in the development of a biocontrol solution is understanding normal disease progression and how it is interrupted. The small signaling molecules SA, JA, and ETH have been studied for their involvement in SAR and ISR but they do not act independently within plants. Crosstalk from multiple additional signals can promote or antagonize the effects of these signaling molecules on plant resistance. It is becoming apparent that ABA is a critical factor involved in modulating plant defenses that may be manipulated in different ways by pathogens and beneficial microbes. The study of ABA as it relates to biotic stress is relatively new, and there is very little known about how biocontrol bacteria affect this important hormone. However, a crucial role for ABA was recently discovered in the growth promotion of tomato by *Bacillus megaterium* (Porcel et al., 2014). Using the interaction between EA105, rice, and *M. oryzae* as a model system, we sought to investigate how the beneficial bacteria and the pathogenic fungus may be manipulating ABA to influence the susceptibility of rice to blast.

Since elevated ABA levels have been shown to increase rice susceptibility to *M. oryzae* and *X. oryzae* (Koga et al., 2004; Jiang et al., 2010; Yazawa et al., 2012; Xu et al., 2013), the effect of EA105 and 70-15 on the expression of key ABA biosynthesis gene *NCED3* was examined. As expected, 70-15 treatment up-regulated *NCED3* in rice, while EA105 had very little effect. Interestingly, when rice plants were treated with EA105 prior to infection with spores, there was no longer an up-regulation of *NCED3*, potentially indicating that EA105 is preventing spores from increasing ABA biosynthesis in rice. When plants were treated with ABA, which also induces *NCED3* expression, and EA105, the up-regulation of *NCED3* persisted. EA105 was able to prevent spores but not exogenous ABA from up-regulating *NCED3* in rice, indicating that spores are affecting plants in ways that differ from ABA alone. In this aspect, EA105 was not able to counter the effects of ABA, yet it did attenuate the effects of spores.

In contrast to the expression of ABA biosynthesis gene *NCED3*, actual ABA concentrations were examined in plants with the same treatments and no differences were found. While it has been shown that elevated levels of ABA increase susceptibility to *M. oryzae* during very early stages of infection (Yazawa et al., 2012), it is possible that ABA once again becomes important in later stages of disease progression. For instance, in sugar beets the highest levels of ABA were not seen until 15 days post-infection, when the fungal pathogen switched to the necrotic phase of infection (Schmidt et al., 2008). *M. oryzae* is also a hemi-biotrophic fungus, and it is possible that ABA levels in rice only reach their maxima in later stages of necrosis.

The discrepancy between *NCED3* expression and ABA concentration has been shown previously (Priest et al., 2006; Ye et al., 2012). Beyond biosynthesis, ABA concentrations are also influenced by catabolism, activation and inactivation, transport, compartmentalization, and inhibition of signal transduction. In rice, primers were designed based on putative genes and both were highly responsive to exogenous ABA treatment. Similarly to what was seen with *NCED3*, spores increased expression of the putative β-glucosidase, potentially involved in activating ABA, in rice roots while the presence of EA105 prevented this.

Yet another factor to consider is the degree by which actual ABA concentrations contribute to ABA-mediated responses. ABA signaling pathways are intersected by multiple pathways including GA, SA, JA, and ET (Robert-Seilaniantz et al., 2011; Denance et al., 2013; Yang et al., 2013; Meguro and Sato, 2014), all of which can affect ABA-mediated responses without directly altering ABA concentrations. Regulation within ABA signaling, such as inactivation and binding of PP2Cs to the SnRK2s, also effect ABA-mediated responses, as does the availability of ABA receptors. Yazawa et al. (2012) noticed that in the mutant *OsABI* line, impaired in ABA signaling, there were actually higher levels of ABA though they still saw fewer lesions (Yazawa et al., 2012). It is an important observation that lesions were reduced in plants which had elevated levels of ABA, but were impaired in a later step in ABA signaling, indicating that the actual ABA concentrations may not be as crucial as ABA perception and signaling in ABA-mediated resistance. Corroborating this, we saw that EA105 affected ABA gene expression in rice and *M. oryzae* without altering ABA concentration.

A hormone such as ABA that is involved in a multitude of normal developmental and stress-induced responses in plants must be tightly regulated. Each process for regulating the amount of active ABA, such as inactivation or catabolism, has multiple genes that can be triggered by different stimuli. ABA responses and ABA signal transduction can differ based on the type of stress, with particular differences having been found between abiotic and biotic stress (Kim, 2012; Ye et al., 2012). ABA was originally studied for its role in abiotic stress response, and most of what is known has been determined through experimenting with drought, temperature, and salt stress (Lim et al., 2012) while there is comparatively much less known about the role of ABA in biotic stress responses. There were only minimal expression changes to ABA-catabolism gene *OsABA8ox1*, ABA receptor *RCAR5*, *OsMPK1*, or *Rab25*. It is possible that these genes are not involved in this particular biotic stress situation, while other ABA-related genes that were not tested may be playing a role.

Based on expression of *NCED3* and the putative rice β-glucosidase, it is possible that modulating ABA signaling in plants may be a contributing factor to how EA105 reduces blast lesions. It has been established previously that ABA plays a role in plant susceptibility to pathogens. However, ABA may be playing a dual role by not only increasing susceptibility in plants, but also by directly promoting virulence in *M. oryzae*. We have shown that 70-15 produced ABA during vegetative growth and during spore formation. Exogenous ABA accelerated both spore germination and appressoria formation in 70-15, both of which are necessary for virulence. *M. oryzae* may be producing its own ABA as well as utilizing the increased ABA produced in plants to enhance pathogenicity. ABA is a growth regulator in plants, and may also regulate growth in fungi. To determine if the effect of ABA on 70-15 germination and appressoria formation was specific, other growth regulators were also tested including auxins, cytokinin, and GA. Only ABA was able to accelerate both germination and appressoria formation. Previously, EA105 has been shown to almost completely prevent the formation of appressoria (Spence et al., 2014a,b). When EA105 and ABA were added together on spores, there was a large reduction in the number of spores forming appressoria when compared to ABA treatment alone. Not only is EA105 interfering with ABA’s ability to accelerate appressoria formation, but this also shows that high levels of ABA (100 μM) are still unable to prevent EA105 from reducing appressoria formation.

While ABA’s role in drought tolerance in plants has driven an extensive body of research on plant ABA biosynthesis and signaling, very little is known about fungal ABA biosynthesis, signaling, or even the functional significance of ABA in fungi. Fungal ABA biosynthesis follows an entirely different pathway, utilizing a different set of enzymes. Using characterized ABA biosynthesis gene sequences from *B. cinerea*, we were able to identify a gene in *M. oryzae*, orthologous to ABA4, which appears to significantly reduce the concentration of ABA synthesized by *M. oryzae* spores and mycelia. Not surprisingly, this mutant suffers growth defects and phenotypic abnormalities. However, the most striking characteristic of this ABA4 knockout mutant is its inability to properly form lesions on rice, supporting our hypothesis that not only plant ABA, but also endogenous fungal ABA may be required for infection. Further, adding an un-biologically high level of exogenous ABA (100 μM) only partially restored the wild-type phenotype, indicating that internal fungal ABA biosynthesis and intermediate signaling events, rather than absolute ABA content, may be a crucial part of the infection process. When examining germination and appressoria formation in an isolated, *in vitro* assay, exogenous ABA fully restored the defects of the ABA4 mutant. The process of plant infection is a far more complex and integrated system than germination or appressoria formation alone, which may explain why exogenous ABA can fully rectify deficiencies in those two isolated processes but not in plant infection as a whole.

Another interesting and unexpected set of data has stemmed from the deletion of an *M. oryzae* GPCR involved in ABA signaling. The GPCR deletion mutant sporulated faster and formed bigger lesions than the parental strain, but contained levels of ABA similar to the ABA4 mutant, which was about half the level of the parental strain. Thus, the two mutants behave oppositely yet both have reduced levels of ABA. Again, this points toward a conclusion that absolute ABA levels may not directly determine outcomes, but rather ABA signaling, possibly during the intermediate steps of ABA biosynthesis, might have a larger impact. The complexity and multifaceted nature of ABA in plants has led to elaborate checks and balances to regulate ABA-mediated effects. Fungi are likely to have similar systems in place to control whether ABA is in an active or inactive state, as well as when it is perceived, and when and how the signals are transduced. However, this is an unexplored area and should be the focus of future efforts, as it could shed light on the pathogenesis of economically important phytopathogens.

## Author Contributions

HB conceived the research. CS conducted the experiments and drafted the manuscript. HB provided suggestions and improvements on the manuscript. VL performed Southern blot analysis and contributed to the manuscript. ND provided ideas and guidance in addition to improving the manuscript. All authors read and approved the manuscript.

## Conflict of Interest Statement

The authors declare that the research was conducted in the absence of any commercial or financial relationships that could be construed as a potential conflict of interest.
