# Supplementary material for: Crucial Roles of Abscisic Acid Biogenesis in Virulence of Rice Blast Fungus Magnaporthe oryzae
Source: Front Plant Sci. 2015 Dec 1;6:1082. doi: 10.3389/fpls.2015.01082 (PMC4664623; doi:10.3389/fpls.2015.01082)
Supplement: Supplementary file 1 [file Presentation_1.PPTX]

## Slide 1
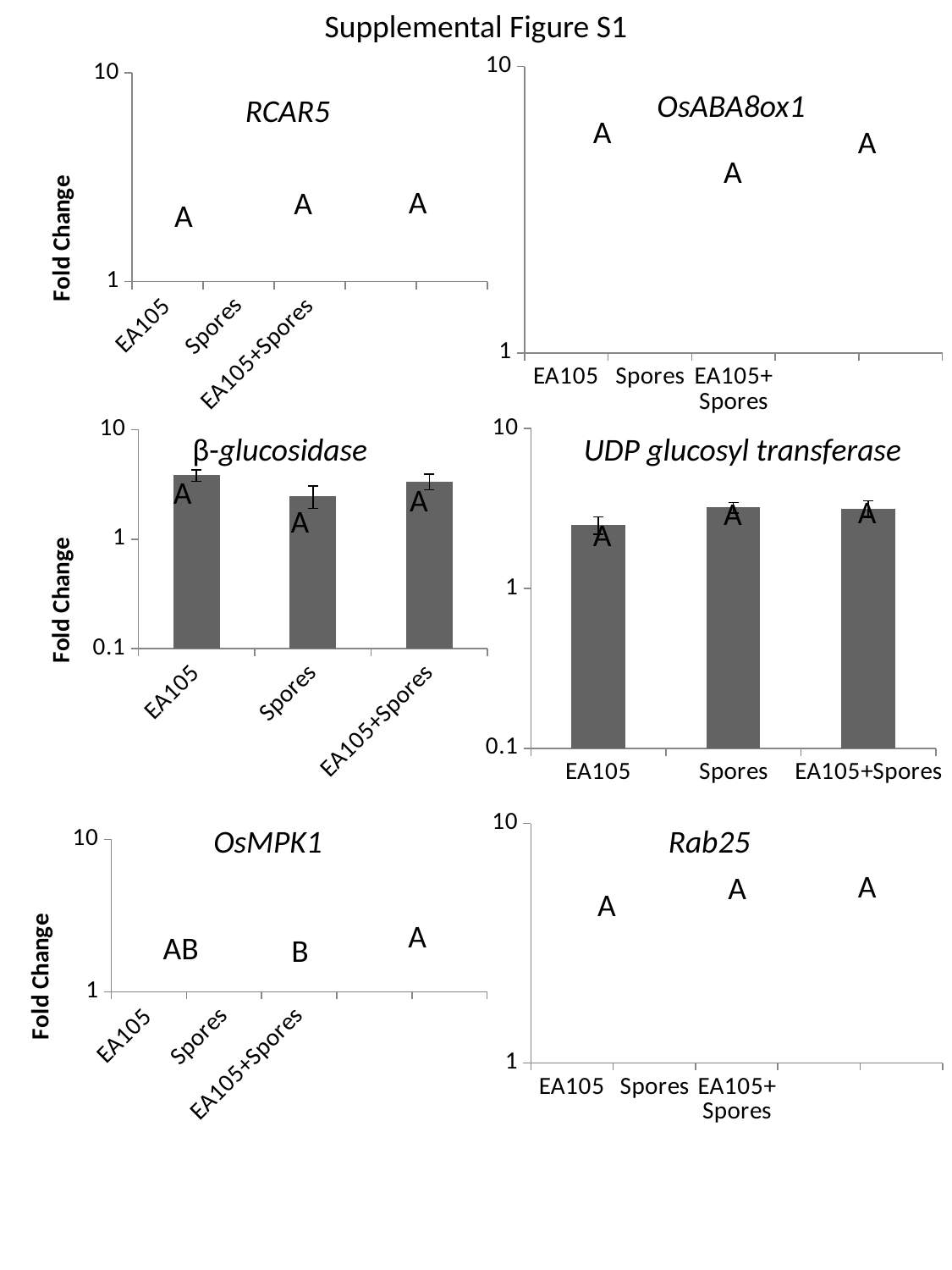

Supplemental Figure S1
### Chart
| Category | |
|---|---|
| EA105 | 3.369495603483891 |
| Spores | 2.093100124668083 |
| EA105+Spores | 2.987428083143262 |
### Chart
| Category | |
|---|---|
| EA105 | 1.466634541845184 |
| Spores | 1.775129260115155 |
| EA105+Spores | 1.706384835989203 |OsABA8ox1
RCAR5
A
A
A
A
A
A
### Chart
| Category | |
|---|---|
| EA105 | 2.494522148468051 |
| Spores | 3.206766631252963 |
| EA105+Spores | 3.16027635887018 |
### Chart
| Category | |
|---|---|
| EA105 | 3.84487422157944 |
| Spores | 2.478395459823534 |
| EA105+Spores | 3.367526698813578 |β-glucosidase
UDP glucosyl transferase
A
A
A
A
A
A
### Chart
| Category | |
|---|---|
| EA105 | 2.179758046930235 |
| Spores | 2.576919291260001 |
| EA105+Spores | 2.747906560669377 |OsMPK1
Rab25
### Chart
| Category | |
|---|---|
| EA105 | 1.173016159212495 |
| Spores | 1.060480863047976 |
| EA105+Spores | 1.398517548995283 |A
A
A
A
AB
B

## Slide 2
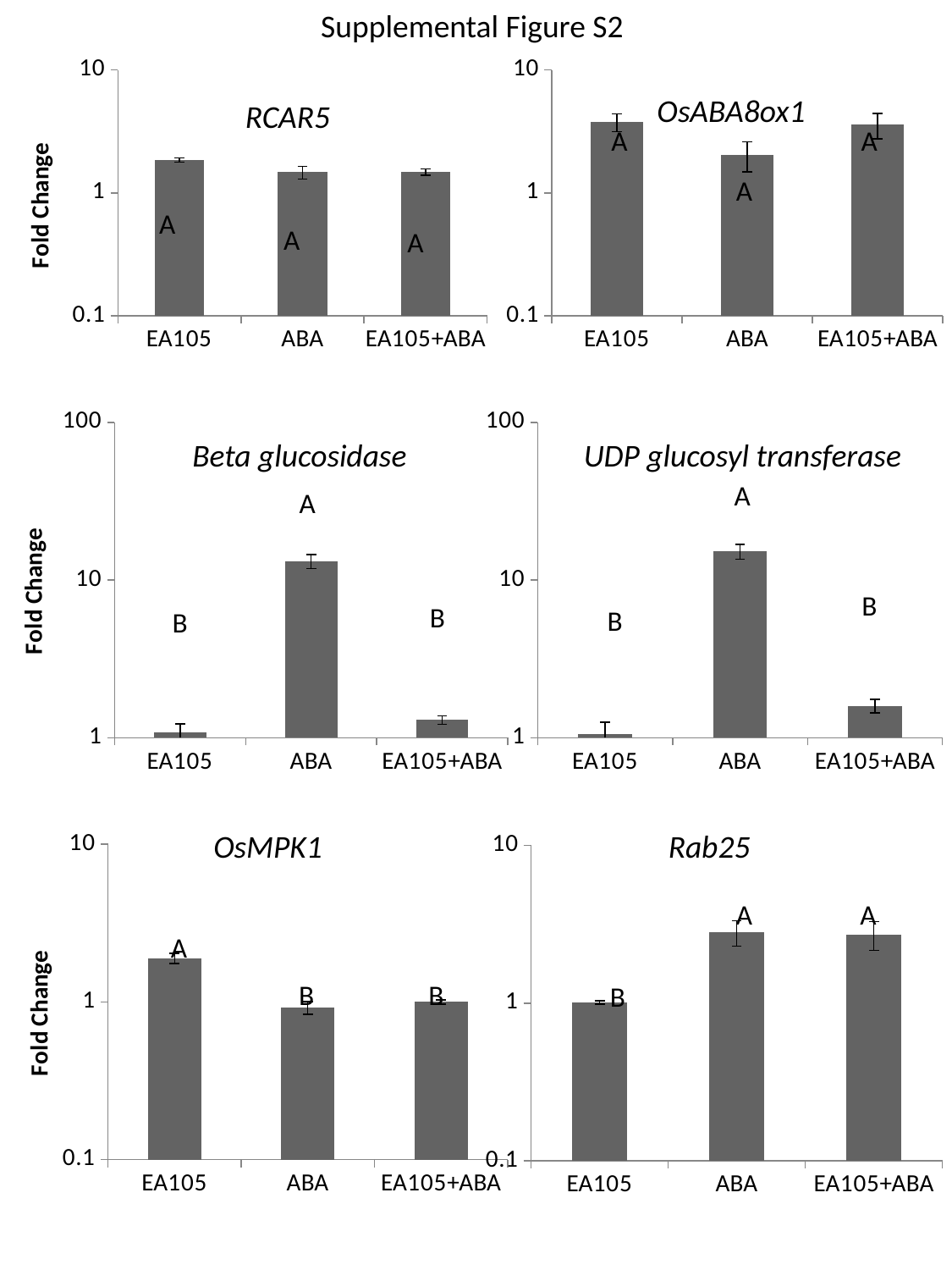

Supplemental Figure S2
### Chart
| Category | |
|---|---|
| EA105 | 3.763499920877713 |
| ABA | 2.048893474804898 |
| EA105+ABA | 3.596775405172969 |
### Chart
| Category | |
|---|---|
| EA105 | 1.852657256890342 |
| ABA | 1.475584289546074 |
| EA105+ABA | 1.480273598650323 |OsABA8ox1
RCAR5
A
A
A
A
A
A
### Chart
| Category | |
|---|---|
| EA105 | 1.083806312547781 |
| ABA | 13.13381329524032 |
| EA105+ABA | 1.29517160995851 |
### Chart
| Category | |
|---|---|
| EA105 | 1.055682032654747 |
| ABA | 15.19686055310215 |
| EA105+ABA | 1.596110815662495 |Beta glucosidase
UDP glucosyl transferase
A
A
B
B
B
B
OsMPK1
Rab25
### Chart
| Category | |
|---|---|
| EA105 | 1.891541133983334 |
| ABA | 0.920623835369181 |
| EA105+ABA | 1.000270843479107 |
### Chart
| Category | |
|---|---|
| EA105 | 1.01301421853529 |
| ABA | 2.824012759029388 |
| EA105+ABA | 2.729184184030234 |A
A
A
B
B
B

## Slide 3
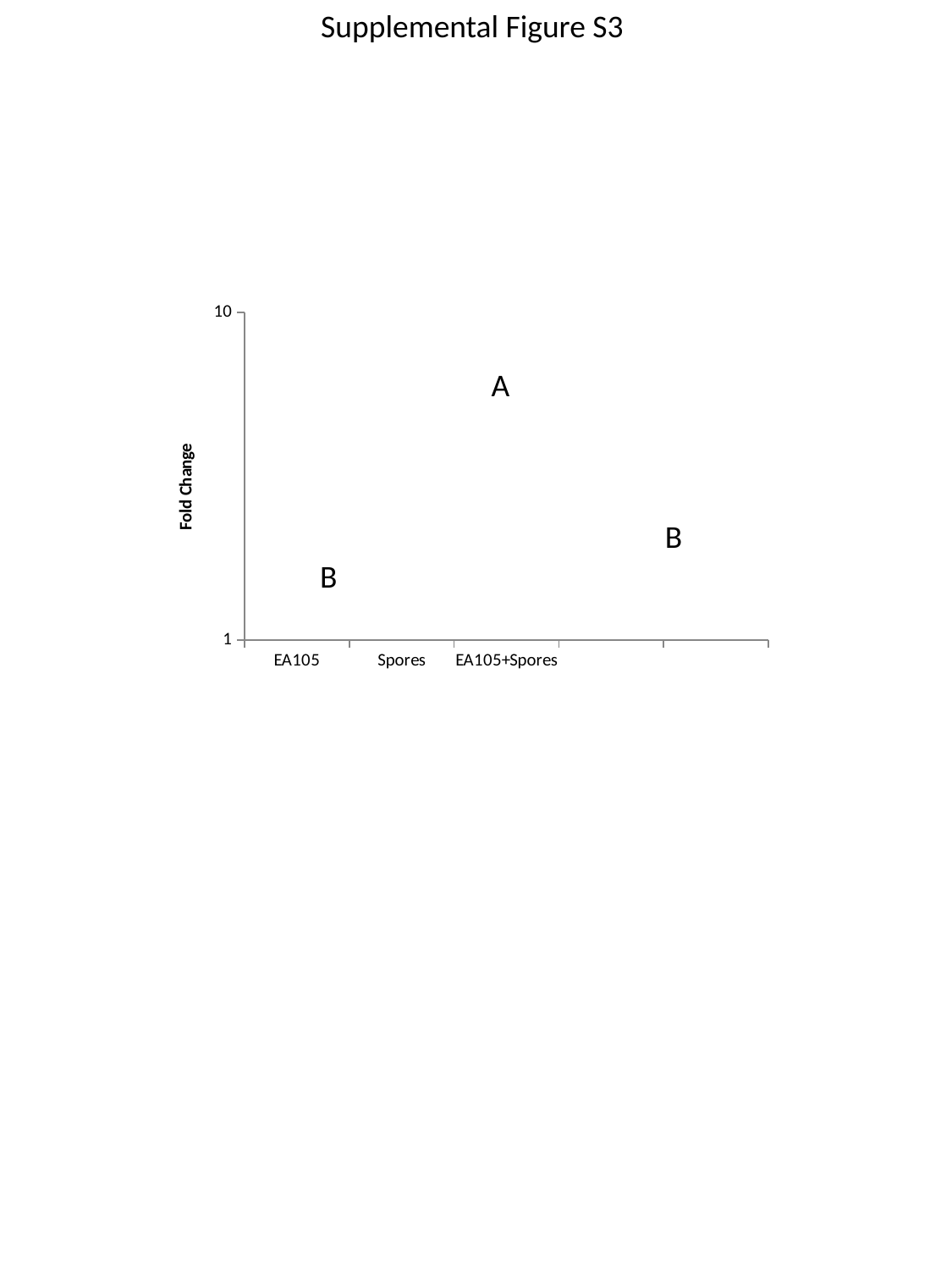

Supplemental Figure S3
### Chart
| Category | |
|---|---|
| EA105 | 1.404219717798737 |
| Spores | 23.85754233311427 |
| EA105+Spores | 2.739278325819232 |A
B
B

## Slide 4
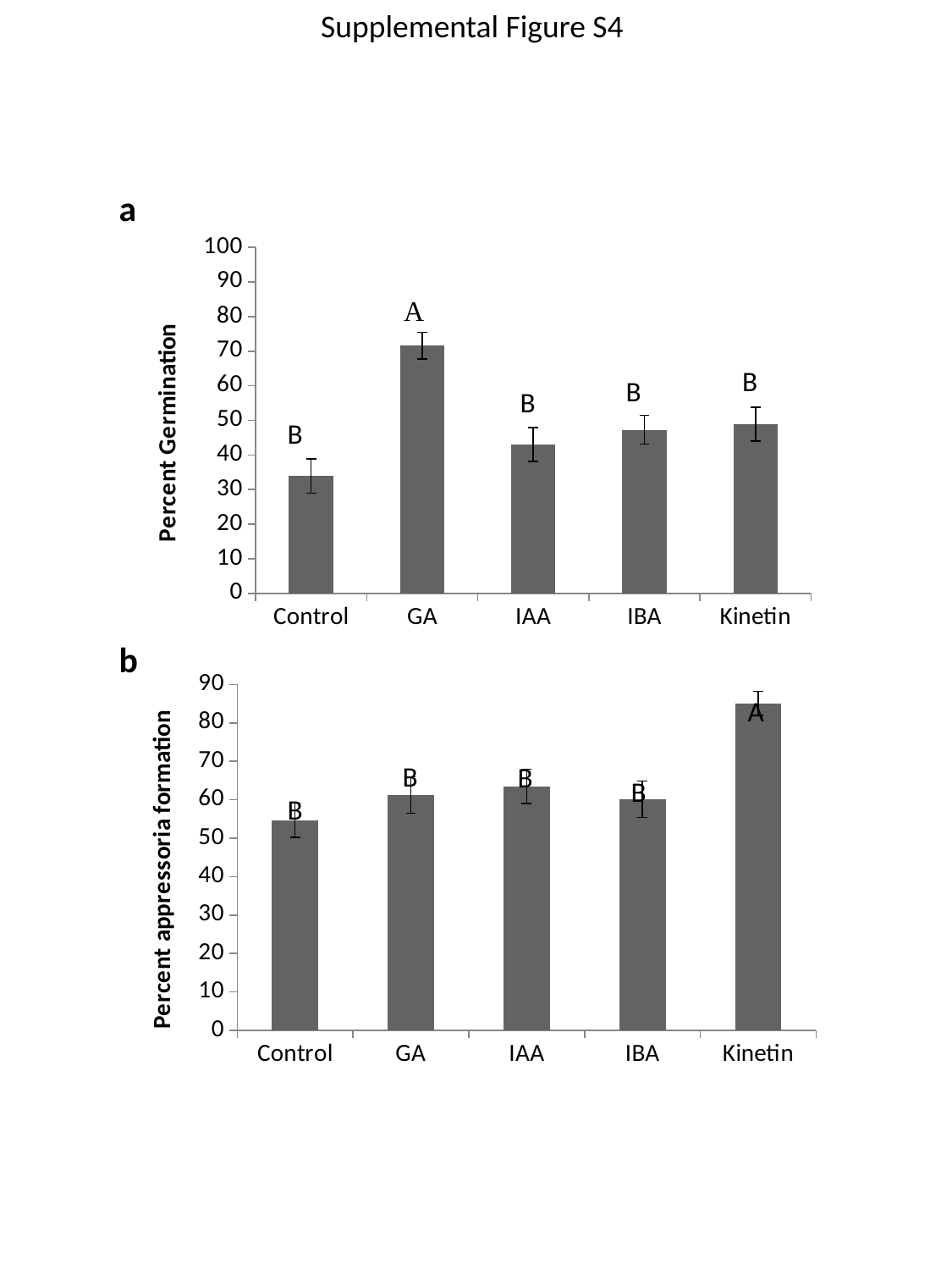

Supplemental Figure S4
a
### Chart
| Category | |
|---|---|
| Control | 33.90313390313391 |
| GA | 71.61654135338337 |
| IAA | 43.002544529262 |
| IBA | 47.26277372262778 |
| Kinetin | 48.8888888888889 |b
### Chart
| Category | |
|---|---|
| Control | 54.6972860125261 |
| GA | 61.25000000000001 |
| IAA | 63.51648351648345 |
| IBA | 60.14492753623188 |
| Kinetin | 85.05050505050505 |A
B
B
B
B

## Slide 5
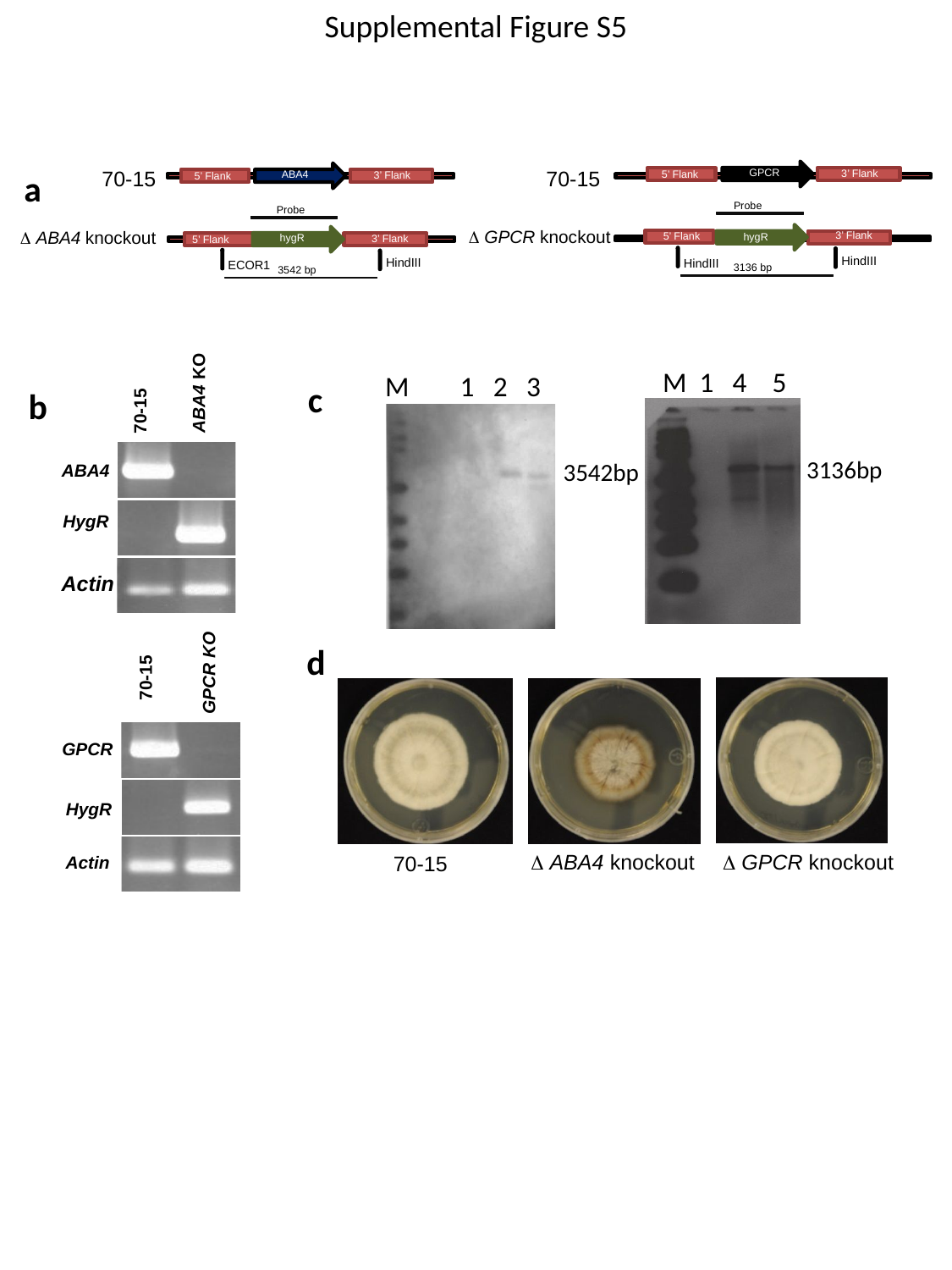

Supplemental Figure S5
70-15
70-15
GPCR
3’ Flank
a
ABA4
3’ Flank
5’ Flank
5’ Flank
Probe
Probe
Δ GPCR knockout
Δ ABA4 knockout
3’ Flank
5’ Flank
hygR
hygR
3’ Flank
5’ Flank
HindIII
HindIII
HindIII
ECOR1
3136 bp
3542 bp
ABA4 KO
M 1 4 5
M 1 2 3
c
b
 70-15
3136bp
3542bp
 ABA4
HygR
Actin
GPCR KO
d
 70-15
GPCR
HygR
Δ GPCR knockout
Δ ABA4 knockout
70-15
Actin

## Slide 6
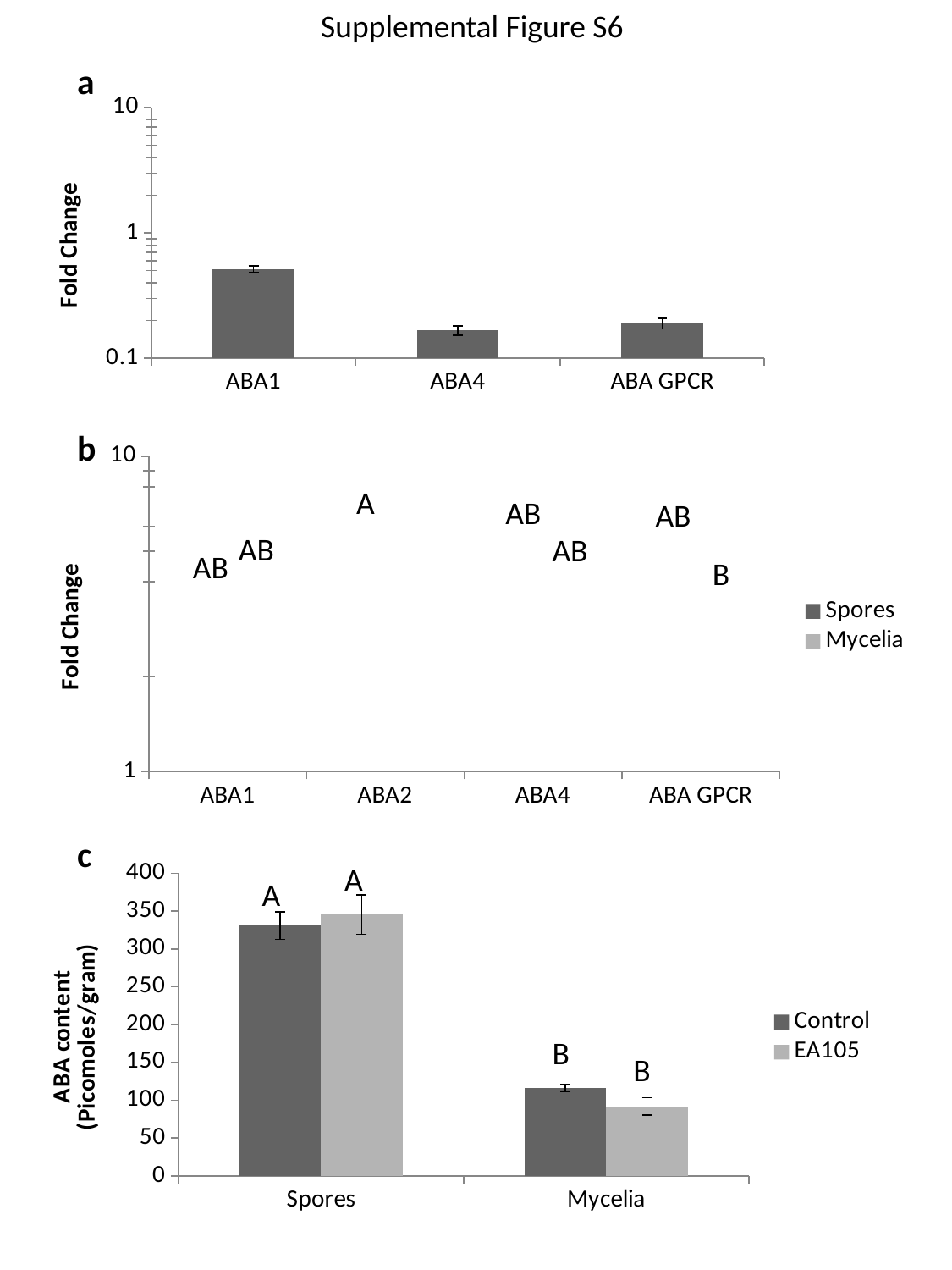

Supplemental Figure S6
a
### Chart
| Category | |
|---|---|
| ABA1 | 0.515675553558998 |
| ABA4 | 0.166675187228038 |
| ABA GPCR | 0.189577222119733 |b
### Chart
| Category | | |
|---|---|---|
| ABA1 | 0.603153384385021 | 0.70957903284152 |
| ABA2 | 0.854830245281888 | 0.0 |
| ABA4 | 0.810572063390644 | 0.75754427178456 |
| ABA GPCR | 0.825935633001322 | 0.578961371410822 |A
AB
AB
AB
AB
AB
B
c
### Chart
| Category | | |
|---|---|---|
| Spores | 330.7561942061983 | 345.2753518010855 |
| Mycelia | 116.0319569813313 | 91.71128697718032 |A
A
B
B

## Slide 7
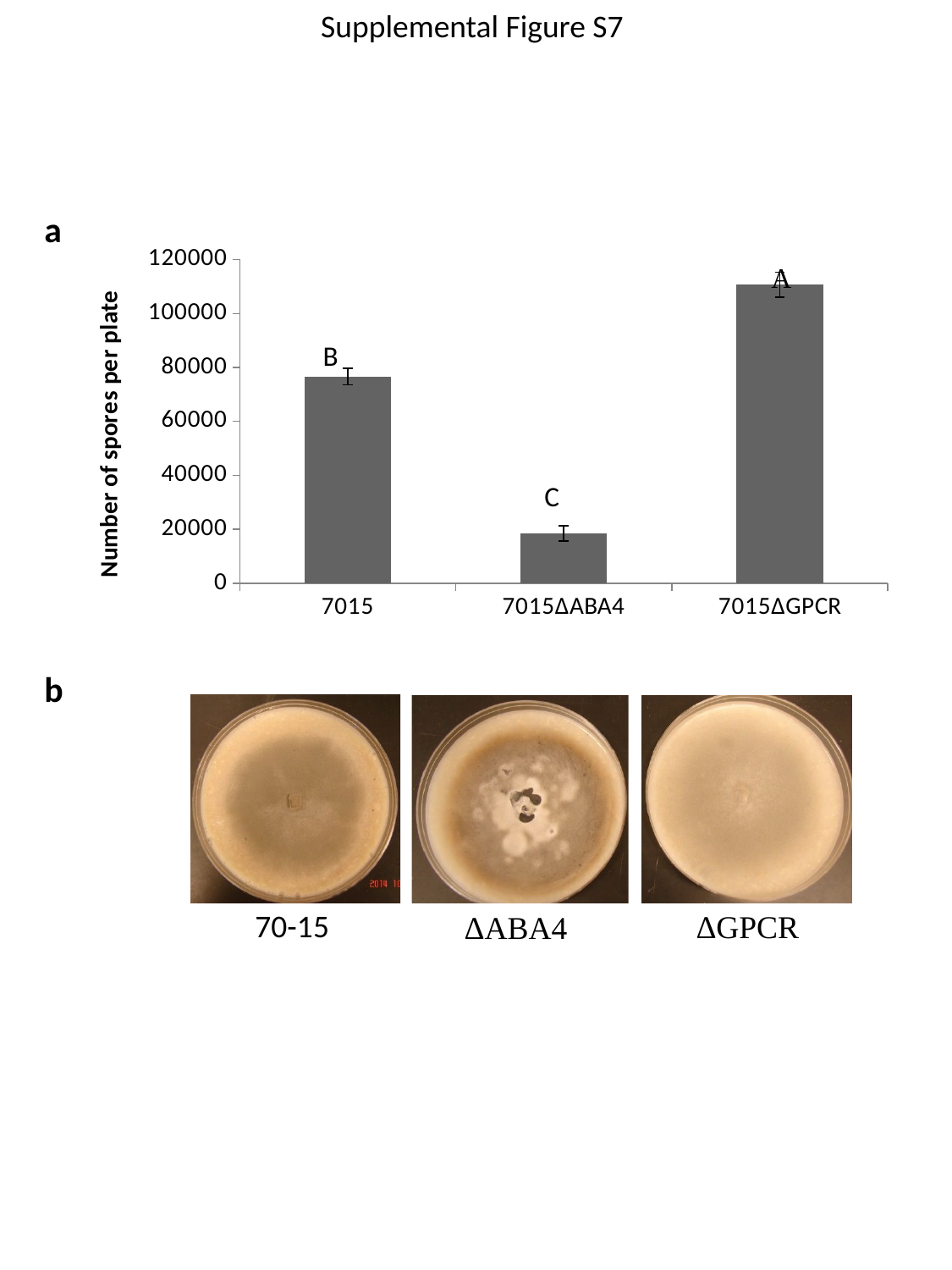

Supplemental Figure S7
### Chart:
| Category | |
|---|---|
| 7015 | 76666.66666666667 |
| 7015ΔABA4 | 18500.0 |
| 7015ΔGPCR | 110666.6666666667 |a
b
70-15
ΔGPCR
ΔABA4

## Slide 8
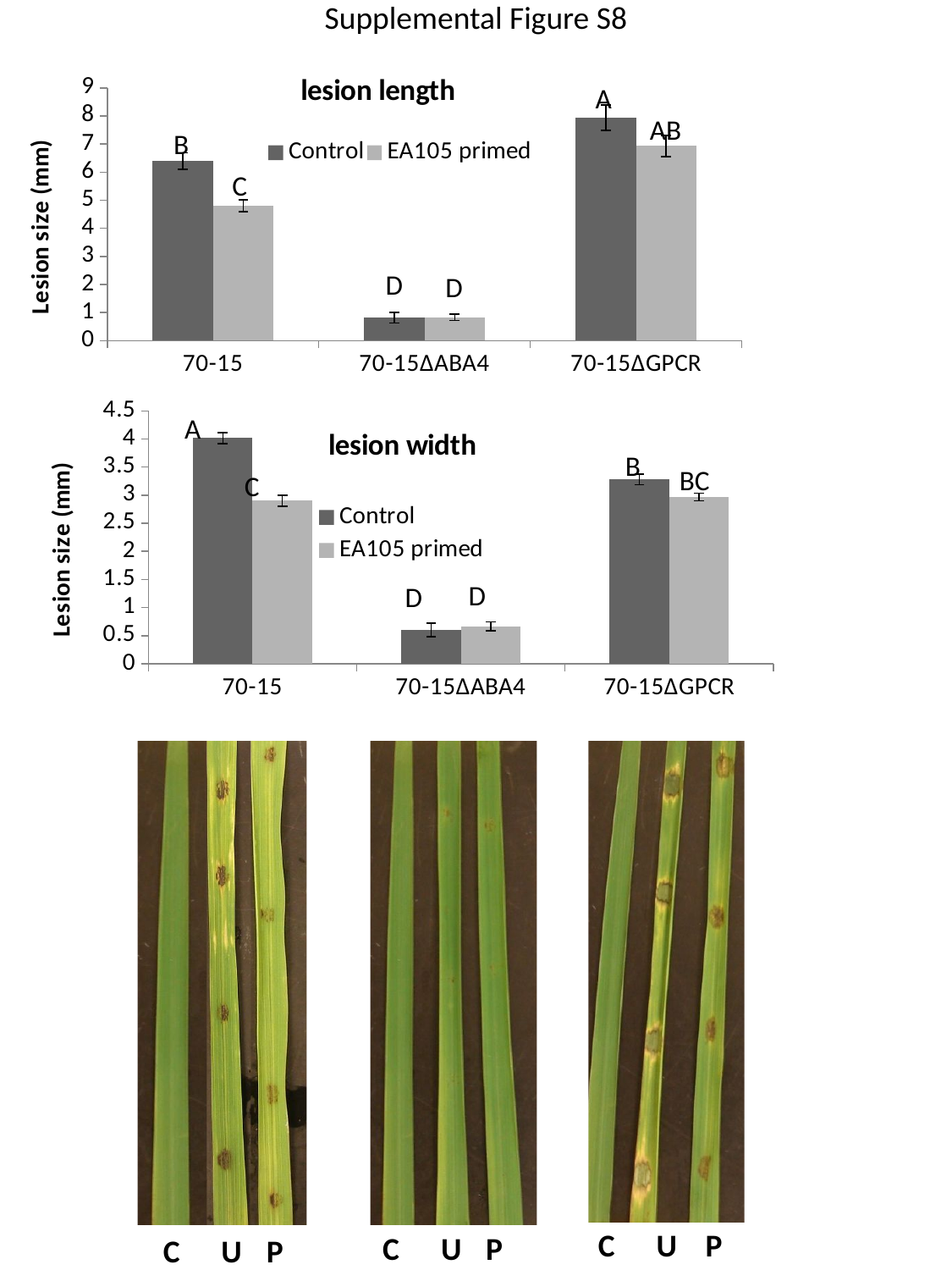

Supplemental Figure S8
### Chart: lesion length
| Category | | |
|---|---|---|
| 70-15 | 6.393617021276595 | 4.798076923076921 |
| 70-15ΔABA4 | 0.822222222222222 | 0.833333333333333 |
| 70-15ΔGPCR | 7.9375 | 6.93333333333334 |A
AB
B
C
D
D
### Chart: lesion width
| Category | | |
|---|---|---|
| 70-15 | 4.01428571428571 | 2.9 |
| 70-15ΔABA4 | 0.6 | 0.666666666666667 |
| 70-15ΔGPCR | 3.28125 | 2.966666666666667 |A
B
BC
C
D
D
C
U
P
C
U
P
C
U
P

## Slide 9
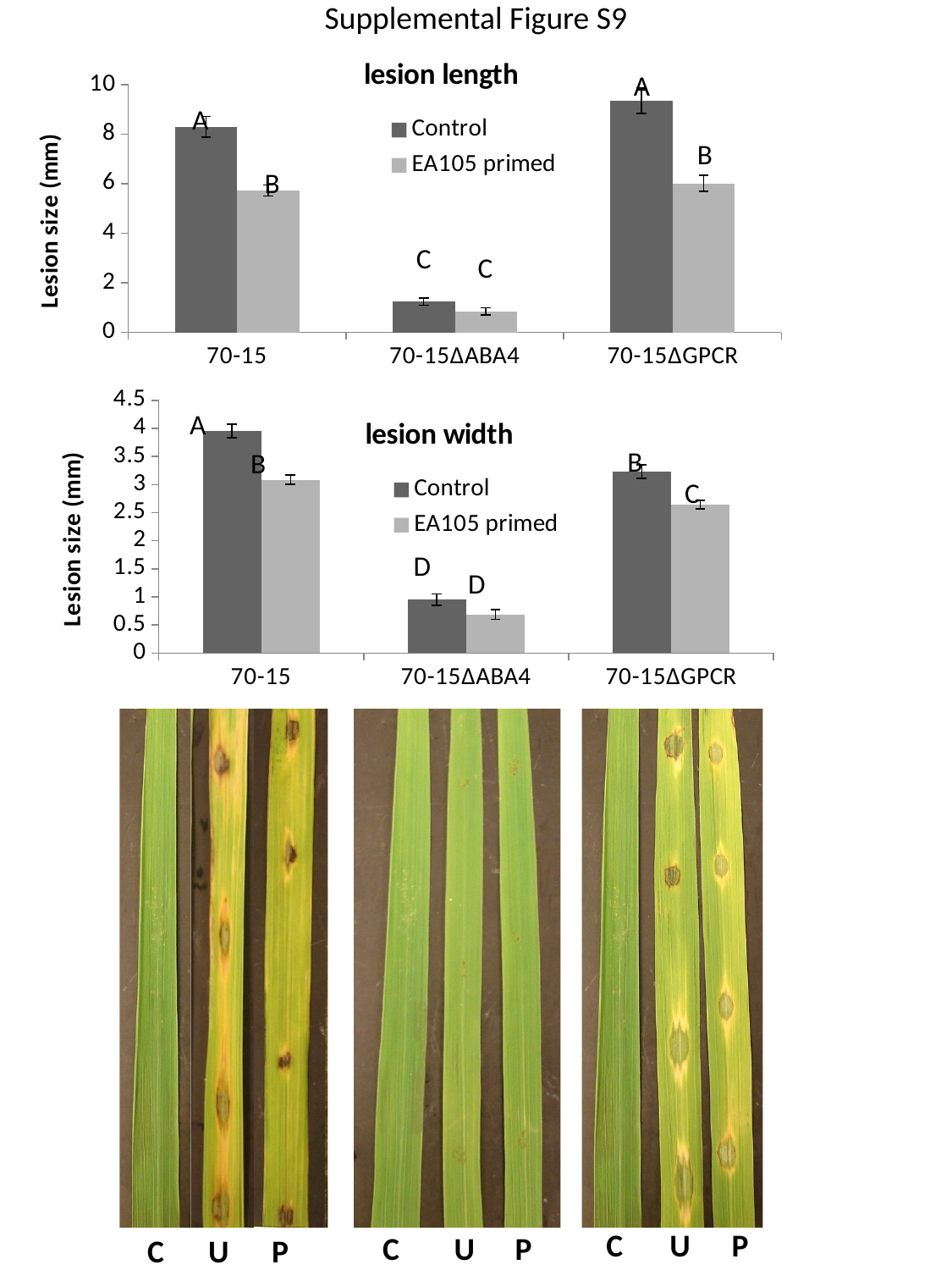

### Chart: lesion length
| Category | | |
|---|---|---|
| 70-15 | 8.294871794871781 | 5.728260869565214 |
| 70-15ΔABA4 | 1.238095238095238 | 0.854166666666666 |
| 70-15ΔGPCR | 9.35 | 6.017857142857141 |Supplemental Figure S9
A
A
B
B
C
C
### Chart: lesion width
| Category | | |
|---|---|---|
| 70-15 | 3.953846153846154 | 3.0875 |
| 70-15ΔABA4 | 0.952380952380952 | 0.6875 |
| 70-15ΔGPCR | 3.233333333333334 | 2.642857142857143 |A
B
B
C
D
D
C
U
P
C
U
P
C
U
P

## Slide 10
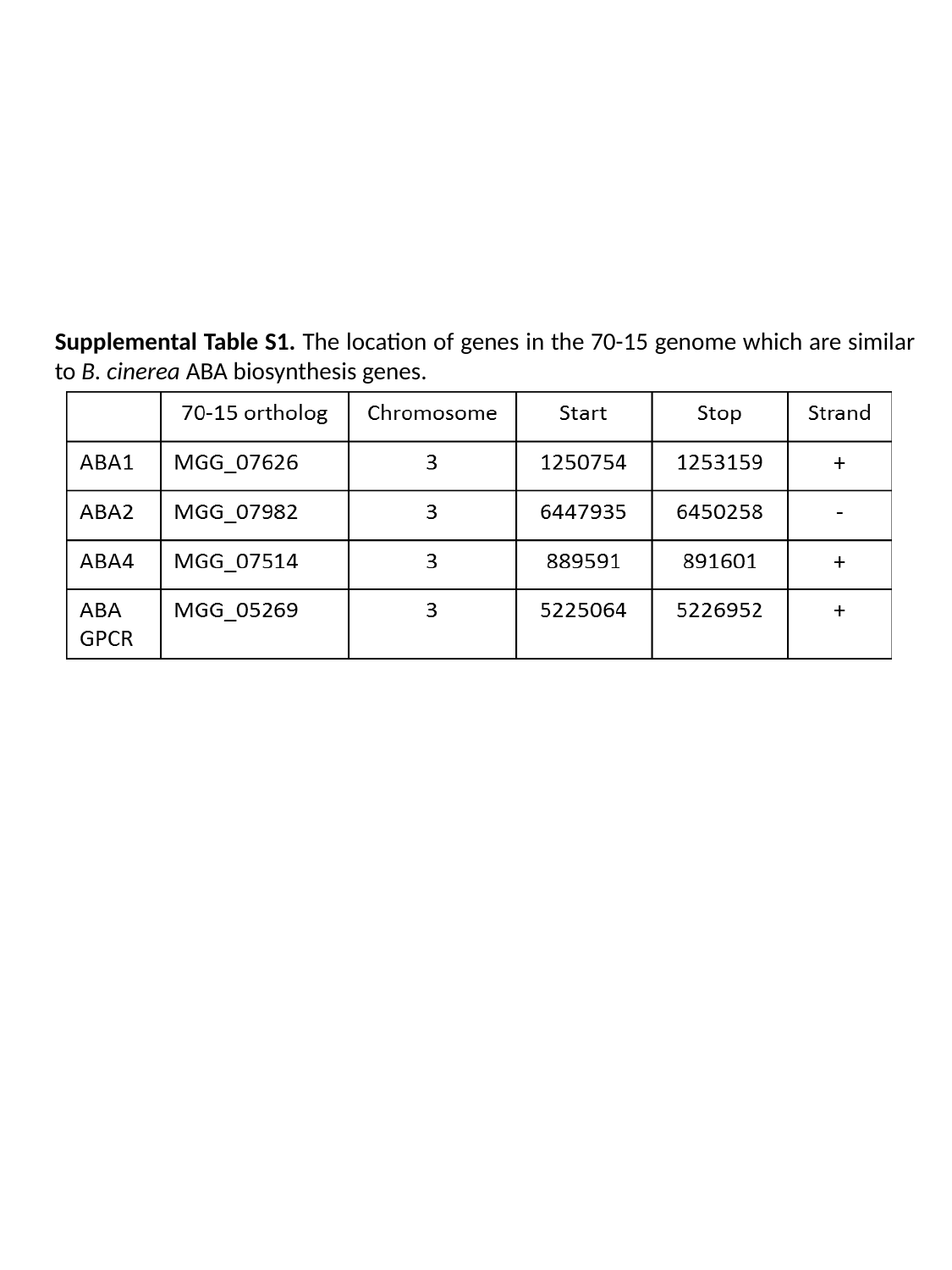

Supplemental Table S1. The location of genes in the 70-15 genome which are similar to B. cinerea ABA biosynthesis genes.

## Slide 11
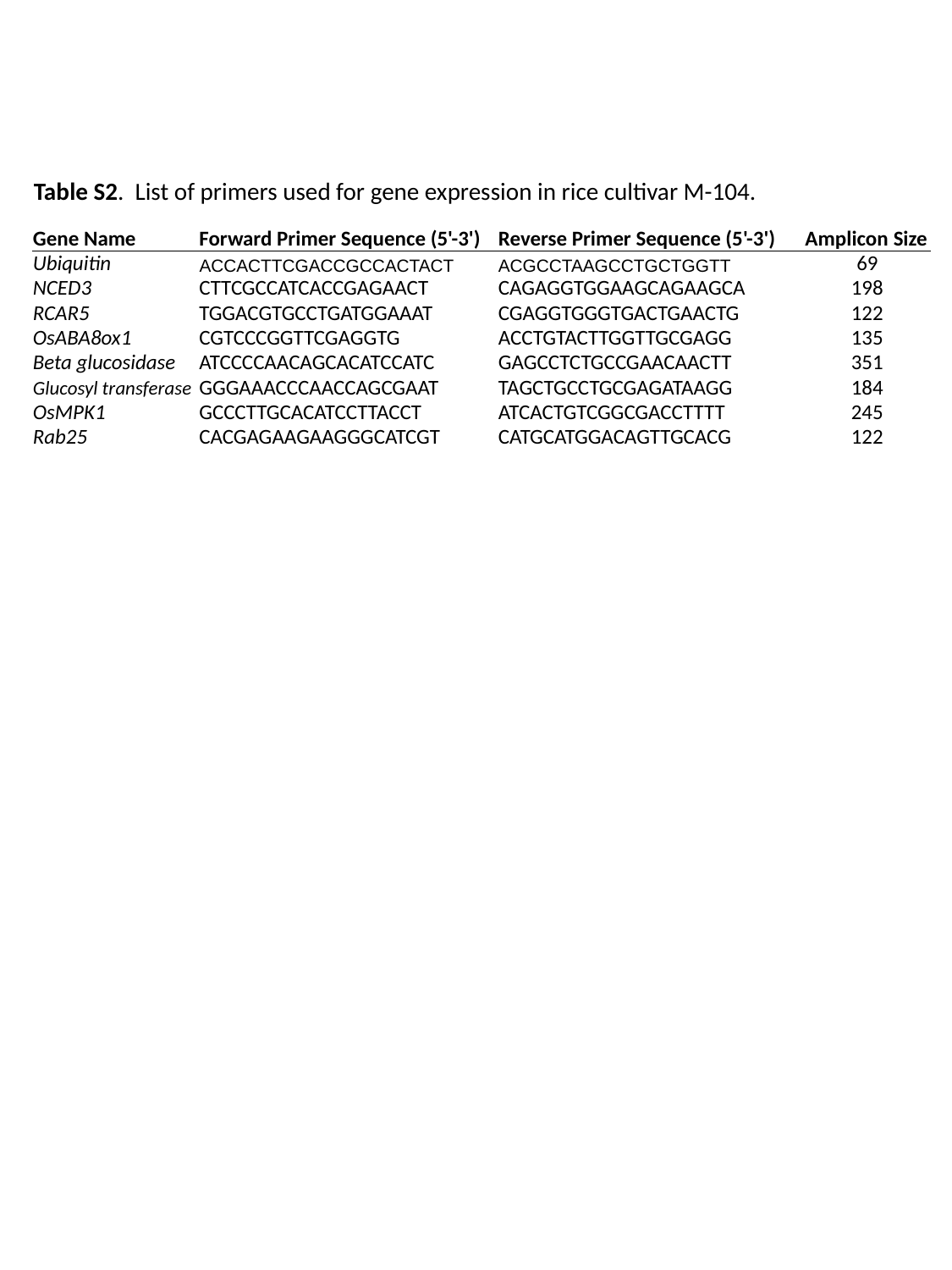

Table S2. List of primers used for gene expression in rice cultivar M-104.
| Gene Name | Forward Primer Sequence (5'-3') | Reverse Primer Sequence (5'-3') | Amplicon Size |
| --- | --- | --- | --- |
| Ubiquitin | ACCACTTCGACCGCCACTACT | ACGCCTAAGCCTGCTGGTT | 69 |
| NCED3 | CTTCGCCATCACCGAGAACT | CAGAGGTGGAAGCAGAAGCA | 198 |
| RCAR5 | TGGACGTGCCTGATGGAAAT | CGAGGTGGGTGACTGAACTG | 122 |
| OsABA8ox1 | CGTCCCGGTTCGAGGTG | ACCTGTACTTGGTTGCGAGG | 135 |
| Beta glucosidase | ATCCCCAACAGCACATCCATC | GAGCCTCTGCCGAACAACTT | 351 |
| Glucosyl transferase | GGGAAACCCAACCAGCGAAT | TAGCTGCCTGCGAGATAAGG | 184 |
| OsMPK1 | GCCCTTGCACATCCTTACCT | ATCACTGTCGGCGACCTTTT | 245 |
| Rab25 | CACGAGAAGAAGGGCATCGT | CATGCATGGACAGTTGCACG | 122 |

## Slide 12
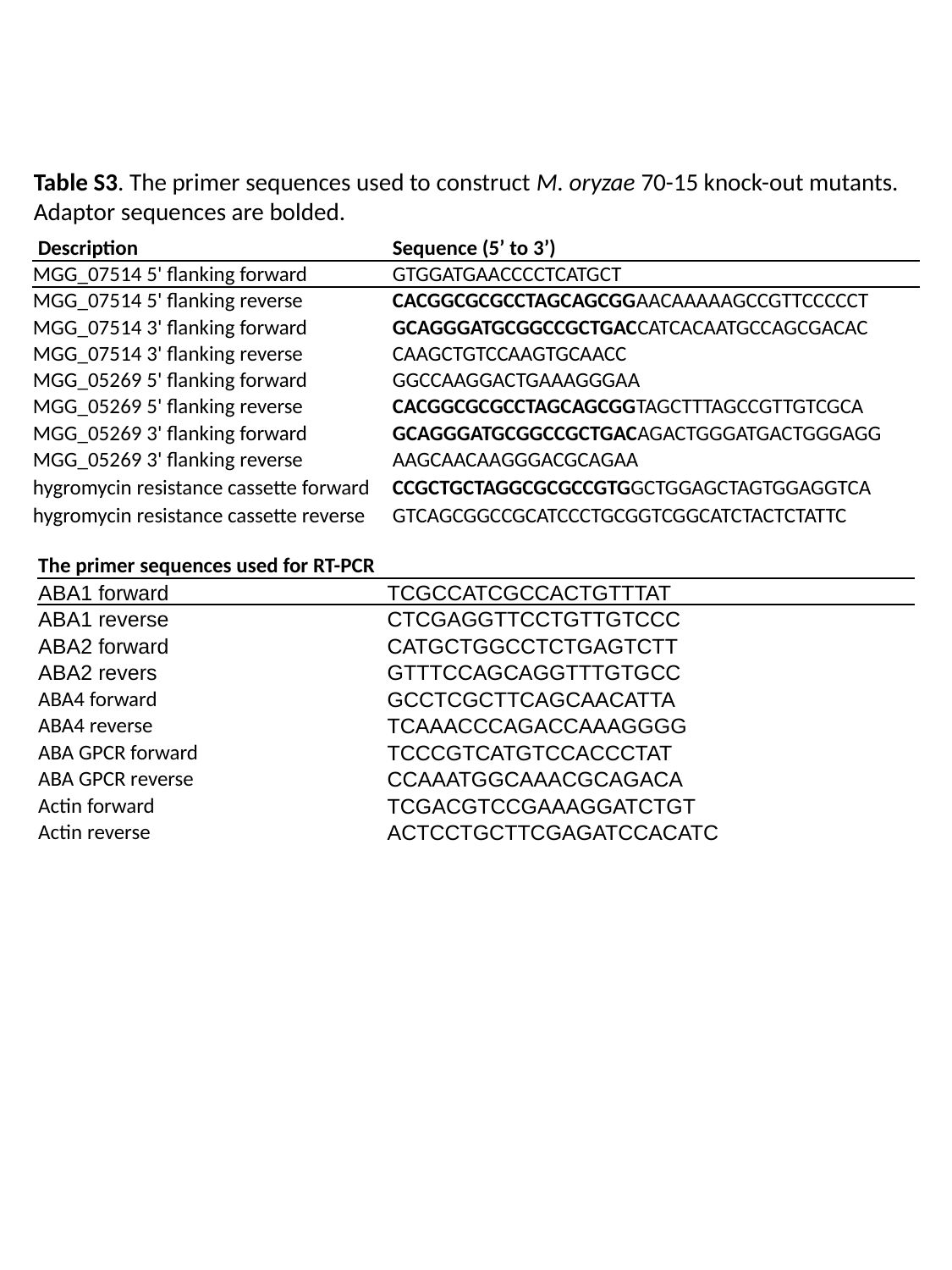

Table S3. The primer sequences used to construct M. oryzae 70-15 knock-out mutants. Adaptor sequences are bolded.
| Description | Sequence (5’ to 3’) |
| --- | --- |
| MGG\_07514 5' flanking forward | GTGGATGAACCCCTCATGCT |
| MGG\_07514 5' flanking reverse | CACGGCGCGCCTAGCAGCGGAACAAAAAGCCGTTCCCCCT |
| MGG\_07514 3' flanking forward | GCAGGGATGCGGCCGCTGACCATCACAATGCCAGCGACAC |
| MGG\_07514 3' flanking reverse | CAAGCTGTCCAAGTGCAACC |
| MGG\_05269 5' flanking forward | GGCCAAGGACTGAAAGGGAA |
| MGG\_05269 5' flanking reverse | CACGGCGCGCCTAGCAGCGGTAGCTTTAGCCGTTGTCGCA |
| MGG\_05269 3' flanking forward | GCAGGGATGCGGCCGCTGACAGACTGGGATGACTGGGAGG |
| MGG\_05269 3' flanking reverse | AAGCAACAAGGGACGCAGAA |
| hygromycin resistance cassette forward | CCGCTGCTAGGCGCGCCGTGGCTGGAGCTAGTGGAGGTCA |
| hygromycin resistance cassette reverse | GTCAGCGGCCGCATCCCTGCGGTCGGCATCTACTCTATTC |
| The primer sequences used for RT-PCR | |
| --- | --- |
| ABA1 forward | TCGCCATCGCCACTGTTTAT |
| ABA1 reverse | CTCGAGGTTCCTGTTGTCCC |
| ABA2 forward | CATGCTGGCCTCTGAGTCTT |
| ABA2 revers | GTTTCCAGCAGGTTTGTGCC |
| ABA4 forward | GCCTCGCTTCAGCAACATTA |
| ABA4 reverse | TCAAACCCAGACCAAAGGGG |
| ABA GPCR forward | TCCCGTCATGTCCACCCTAT |
| ABA GPCR reverse | CCAAATGGCAAACGCAGACA |
| Actin forward | TCGACGTCCGAAAGGATCTGT |
| Actin reverse | ACTCCTGCTTCGAGATCCACATC |
